# Betaine alleviates renal osteodystrophy by stabilizing ABCG1 mRNA to inhibit HMOX1-mediated ferroptosis in BMSCs

**DOI:** 10.1016/j.gendis.2026.102444

**Published:** 2026-09-04

**Authors:** Xinhang Li, Yi Xie, Wenyan Ren, Qing Liu, Qiming Pang, Wenting Liu, Xin Li, Bin He, Dongmei He, Xinyue Wan, Jia Liu, Jie Cai, Baicheng He, Wei Huang, Fang He

**Affiliations:** a Departments of Nephrology, Orthopedics, Urological Metabolism and Immunoecology Laboratory, The First Affiliated Hospital of Chongqing Medical University, Chongqing 400016, China; b Key Laboratory of Biochemistry and Molecular Pharmacology of Chongqing, Chongqing Medical University, Chongqing 400016, China; c Department of Pharmacy, Beijing Anzhen Nanchong Hospital, Capital Medical University & Nanchong Central Hospital, Nanchong, Sichuan 637000, China; d Department of Pharmacy, The Second Clinical Medical College of North Sichuan Medical College, Nanchong, Sichuan 637000, China; e Nanchong Key Laboratory of Individualized Drug Therapy, Nanchong, Sichuan 637000, China; f Department of Orthopaedics, Xiangya Hospital, Central South University, Changsha, Hunan 410008, China; g National Clinical Research Center for Geriatric Disorders, Xiangya Hospital, Changsha, Hunan 410008, China; h Department of Pharmacology, School of Pharmacy, Chongqing Medical University, Chongqing 400016, China; i The Children's Hospital of Chongqing Medical University, Chongqing 400014, China

**Keywords:** ABCG1, Betaine, Bone marrow mesenchymal stem cells, Chronickidney disease, Chronic kidney disease–mineral and bone disorder, Ferroptosis, Renal osteodystrophy

## Abstract

Renal osteodystrophy is a debilitating complication of chronic kidney disease characterized by deteriorated bone microarchitecture and impaired bone formation, leading to increased fracture risk. Despite the pressing need for anabolic therapies, the cellular mechanisms by which uremic stress compromises osteoprogenitor function remain poorly defined. Here, metabolomic profiling of renal osteodystrophy patient cohorts revealed a profound depletion of circulating betaine. We show that betaine supplementation not only rescued the osteogenic differentiation of bone marrow-derived mesenchymal stem cells challenged with the uremic toxin indoxyl sulfate but also ameliorated skeletal deterioration in a rat model of chronic kidney disease. Mechanistically, we demonstrated that indoxyl sulfate triggered ferroptosis in bone marrow-derived mesenchymal stem cells—characterized by iron-dependent lipid peroxidation and aberrant HMOX1 up-regulation—thereby arresting osteogenesis. Betaine mitigates this ferroptotic stress through a novel post-transcriptional mechanism. Specifically, betaine down-regulated the RNA-binding protein hnRNP A1, preventing the recruitment of the CNOT1 deadenylase complex. This inhibition stabilized *Abcg1* mRNA, leading to restored ABCG1 expression. Functional perturbation studies confirmed that the ABCG1–HMOX1 axis was indispensable for the anti-ferroptotic and osteoprotective effects of betaine. Collectively, our findings delineate a Betaine–hnRNP A1/CNOT1–ABCG1–HMOX1 signaling cascade that links metabolic deficiency to ferroptosis-driven osteogenic failure, positioning betaine as a promising anabolic therapeutic strategy for renal osteodystrophy.

## Introduction

Chronic kidney disease (CKD) has emerged as a major global public health crisis, affecting over 10% of the adult population worldwide.^1^^,^^2^ Characterized by irreversible structural damage and progressive loss of renal function, CKD precipitates a range of systemic complications. Prominent among these is chronic kidney disease–mineral and bone disorder (CKD-MBD), a systemic syndrome that disrupts mineral and bone metabolism.^3^ Its specific skeletal manifestation, renal osteodystrophy (ROD), is defined by impaired bone turnover, microarchitectural deterioration, and compromised biomechanical strength. As renal function declines toward end-stage renal disease, the prevalence of ROD escalates dramatically, predisposing patients to a significantly heightened risk of fractures and all-cause mortality.^4^, ^5^, ^6^, ^7^ This imposes a substantial clinical burden that remains inadequately addressed by current therapeutic strategies.

Current management of ROD relies predominantly on maintaining mineral homeostasis by regulating serum parameters such as calcium, phosphorus, and parathyroid hormone.^3^ While this “passive regulation” approach may restore serological balance, it fails to actively promote bone regeneration or rescue compromised bone microstructure—particularly in low-turnover bone disease where anabolic capacity is severely blunted.^8^^,^^9^ This persistent therapeutic impasse highlights a fundamental gap in our understanding of the intrinsic cellular mechanisms governing bone formation within the pathological milieu of CKD.

Traditional research has predominantly focused on the passive responses of mature bone cells to mineral disturbances.^10^, ^11^, ^12^, ^13^ However, emerging evidence suggests that functional deficits in bone marrow-derived mesenchymal stem cells (BMSCs) and bone loss occur in early CKD, preceding overt systemic mineral disturbances.^14^, ^15^, ^16^, ^17^ As the fundamental progenitors for skeletal regeneration, BMSCs orchestrate new bone formation via self-renewal and osteogenic lineage differentiation.^18^^,^^19^ Therefore, early impairment of their function may fundamentally disrupt the effective supply of osteoblasts, which could represent a critical upstream event in the pathogenesis of low-turnover bone disease. In this process, the accumulation of uremic toxins acts as a key factor in reshaping the CKD bone microenvironment and damaging BMSCs. Notably, indoxyl sulfate (IS), a prototypical protein-bound toxin that accumulates early and is refractory to conventional dialysis, has been identified as a critical culprit in CKD-associated complications, including bone disease.^20^, ^21^, ^22^, ^23^ Although IS is known to suppress BMSC osteogenesis,^24^^,^^25^ the precise molecular machinery driving this dysfunction remains to be fully elucidated.

In parallel with toxin accumulation, CKD is increasingly viewed as a state of diminished endogenous cytoprotection, where protective metabolites are depleted due to metabolic disruption. Betaine, an essential endogenous osmolyte and primary methyl donor in one-carbon metabolism, exhibits potent antioxidant and anti-inflammatory properties.^26^^,^^27^ Although circulating betaine levels are known to decline with CKD progression,^28^ its specific role in skeletal homeostasis remains underexplored. Emerging evidence from non-uremic contexts suggests betaine exerts a positive regulatory effect on bone metabolism.^29^^,^^30^ Given that IS triggers severe oxidative stress and lipid peroxidation—the biochemical hallmarks of ferroptosis—we hypothesize that the depletion of betaine compromises the stress resilience of osteoprogenitors, thereby exacerbating IS-driven osteogenic failure. However, whether betaine deficiency contributes directly to ROD pathogenesis and whether its supplementation can rescue BMSCs function remain to be elucidated.

In the present study, untargeted metabolomics identified a significant depletion of serum betaine in patients with ROD. Subsequent functional assays demonstrated that betaine supplementation not only ameliorated renal dysfunction and skeletal abnormalities in CKD rats but also effectively protected BMSCs from IS-induced osteogenic arrest. Mechanistically, we delineated a novel signaling axis wherein betaine mitigates IS-driven ferroptosis in BMSCs via post-transcriptional modulation of the heterogeneous nuclear ribonucleoprotein A1 (hnRNP A1)/ATP-binding cassette transporter G1 (ABCG1) axis. Collectively, these findings suggest that targeting this metabolic-molecular pathway represents a promising anabolic strategy for the clinical management of ROD.

## Materials and methods

### Clinical study design and participants

This study was approved by the Institutional Ethics Committee of The First Affiliated Hospital of Chongqing Medical University (Approval No. 2025-1037-01) and conducted in strict accordance with the Declaration of Helsinki. Written informed consent was obtained from all participants before their enrollment. The clinical investigation comprised two phases: a discovery phase using untargeted metabolomics and a validation phase using targeted assays across varying stages of CKD.

#### Discovery cohort (metabolomics analysis)

To screen for metabolic alterations associated with ROD, a total of 26 participants were recruited. This cohort included 14 patients with clinically diagnosed ROD and 12 age- and sex-matched healthy controls. Serum samples from this cohort were subjected to untargeted metabolomic sequencing to identify differentially abundant metabolites. The detailed clinical profiles of the participants in this cohort are shown in Table S1.

#### Validation cohort (targeted quantification)

To validate the identified metabolic changes and assess their correlation with disease progression, an independent cohort of 100 participants was recruited. This cohort was stratified into three groups based on kidney function: i) healthy controls (*n* = 30): Individuals with normal kidney function undergoing routine health examinations; ii) early-to-moderate CKD (Stages 1–3, *n* = 35): Patients with an estimated glomerular filtration rate (eGFR) ≥ 30 mL/min/1.73 m^2^; iii) advanced CKD (Stages 4–5, *n* = 35): Patients with eGFR < 30 mL/min/1.73 m^2^, including those on dialysis. Detailed clinical data for the validation cohort are provided in Table S2.

#### Inclusion and exclusion criteria

Inclusion criteria for CKD patients were as follows: i) diagnosed kidney injury or an eGFR < 90 mL/min/1.73 m^2^ or an eGFR > 90 mL/min/1.73 m^2^ but defined by kidney damage markers persisting for ≥ 3 months (consistent with KDIGO guidelines for CKD stages 1–5); and ii) age ≥ 18 years. For the ROD group in the discovery cohort, the diagnosis was defined according to the KDIGO 2017 Clinical Practice Guideline for CKD-MBD. Specifically, patients were required to present with evidence of bone metabolic abnormalities, which was defined as either abnormal serum parathyroid hormone levels or radiographic evidence of bone disease. Serum parathyroid hormone was measured using a chemiluminescence immunoassay in our hospital laboratory, with an assay-specific reference range of 12–88 pg/mL. Abnormal serum parathyroid hormone was defined as values outside this reference range, including decreased parathyroid hormone levels (< 12 pg/mL) or elevated parathyroid hormone levels (> 88 pg/mL). Radiographic evidence of bone disease included osteoporosis or osteopenia confirmed by bone mineral density measurement (T-score ≤ −1.0). Exclusion criteria across both cohorts included: i) active chronic infections (*e.g.*, hepatitis B, syphilis); ii) recent use (within 3 months) of medications significantly altering bone metabolism (*e.g.*, bisphosphonates, systemic glucocorticoids); iii) malignancies or severe hematological disorders; iv) severe cardiovascular events (NYHA class III–IV); and v) inability to provide informed consent.

#### Clinical sample collection and analysis

##### Metabolomic profiling

Serum samples from the discovery cohort were analyzed using an Agilent 1290 Infinity LC system coupled with an AB Sciex TripleTOF 6600 mass spectrometer (Shanghai Applied Protein Technology Co., Ltd.). Raw data were processed using ProteoWizard for format conversion (Wiff to MzXML) and XCMS for peak integration. Key processing settings included mass accuracy of 10 ppm, peak width of 10–60 s, and bandwidth of 5. After sum-normalization, the R package ropls was used to identify differential metabolites, defined by a Variable Importance in Projection (VIP) score > 1 and statistical significance (*p* < 0.05).

##### Targeted biochemical assays

In the validation cohort, serum levels of IS and betaine were quantified using commercially available enzyme-linked immunosorbent assay (ELISA) kits (Quanzhou Ruixin Biotechnology Co., Ltd., Quanzhou, China). Specifically, IS concentrations were determined using a competitive ELISA kit (JRXW2015166), while betaine levels were measured using a specific ELISA kit (RXWB0187-96). Absorbance was measured at 450 nm for IS and 525 nm for betaine using a microplate reader. All samples were assayed in duplicate.

##### Clinical data

Demographic and biochemical data (*e.g.*, serum creatinine, calcium, phosphate, parathyroid hormone) were retrieved from electronic medical records.

### Cell culture and chemicals

BMSCs were harvested from Sprague–Dawley rats. HEK293, C3H10T1/2, MC3T3-E1, and OCY454 cell lines were acquired from the American Type Culture Collection. Cells were cultured in Dulbecco's modified Eagle medium (DMEM) supplemented with 10% fetal bovine serum, 100 U/mL penicillin, and 100 μg/mL streptomycin at 37 °C under a humidified 5% CO_2_ atmosphere. Primary antibodies against β-Actin (AC038), RUNX2 (A11753), and ABCG1 (A17907) were purchased from ABclonal (Wuhan, China). Primary antibodies against OPN (22952-1-AP), GPX4 (67763-1-Ig), SLC7A11 (26864-1-AP), HMOX1 (10701-1-AP), ACSL4 (22401-1-AP), HNRNPA1 (11176-1-AP), and CNOT1 (14276-1-AP) were obtained from Proteintech (Wuhan, China). Experimental compounds, including IS (P588486-250 mg), betaine (B105554-1 kg), and Ferrostatin-1 (F408509), were acquired from Aladdin. In the subsequent cell experiments, the IS concentrations (125, 250, and 500 μM) were selected based on preliminary screening and literature reports.^25^^,^^31^^,^^32^ Although serum IS levels in advanced CKD patients typically range from 30 to over 100 μM, utilizing higher concentrations *in vitro* is a well-established convention to compensate for the shorter exposure duration compared with chronic *in vivo* accumulation.^25^^,^^31^^,^^32^ Protoporphyrin zinc (Z836208-10 mg) was purchased from Macklin.

### Recombinant adenovirus construction

Recombinant adenoviruses carrying ABCG1 overexpression (AdABCG1) and knockdown (AdsiABCG1) constructs were generated using the AdEasy system. A green fluorescent protein (GFP)-expressing adenovirus was also constructed as a transduction control. The coding sequences of ABCG1 and GFP were amplified by PCR and individually cloned into the adenoviral shuttle vector. Recombinant vectors were transfected into HEK293 cells for viral packaging and titer determination.

### Osteogenic differentiation induction

Osteogenesis was induced by culturing the cells in a standard osteogenic induction medium consisting of β-glycerophosphate (10 mM), dexamethasone (100 nM), and l-ascorbic acid (250 μM). The medium was refreshed every 3 days throughout the induction period.

### Alkaline phosphatase (ALP) staining

BMSCs were seeded in 24-well plates at a density of 2 × 10^4^ cells per well and induced with the osteogenic induction medium for 5 or 7 days. After induction, cells were gently washed twice with phosphate-buffered saline (PBS) and fixed with 4% paraformaldehyde at room temperature for 15 min. ALP activity was detected using a BCIP/NBT Alkaline Phosphatase Color Development Kit (C3206, Beyotime, Shanghai, China) following the manufacturer's instructions. Staining was performed in the dark for 30 min, followed by PBS washes. Whole-well images were acquired using a flatbed scanner (Phentom, China) with Microtek ScanWizard 5 software. High-magnification images of representative fields were captured under an optical microscope (IX53, Olympus, Tokyo, Japan).

### Alizarin red S staining

After 14–21 days of induction, cells were washed with PBS (pH 4.2), fixed with 4% paraformaldehyde for 15 min, and stained with 0.2% Alizarin red S solution (G8550, Solarbio, Beijing, China) at room temperature for 20 min. Unbound dye was removed by extensive washing with distilled water. Mineralized nodules were visualized and imaged using a scanner and optical microscope. For quantification, the stained nodules were dissolved in 10% cetylpyridinium chloride for 30 min, and absorbance was measured at 562 nm using a microplate reader.

### RNA extraction and quantitative real-time PCR

Total RNA extraction from cultured cells was carried out using TRIzol reagent (15596026, Invitrogen, USA). RNA purity and concentration were assessed spectrophotometrically using a NanoDrop One system (Thermo Fisher Scientific, USA). cDNA was synthesized using a reverse transcription kit (RR037A, Takara) and diluted 5- to 10-fold with nuclease-free water to serve as a template for subsequent quantitative PCR analysis. Quantitative real-time PCR was carried out using 2 × SYBR Green qPCR Master Mix Kit (B2120, Selleck, USA) on a Bio-Rad CFX Connect Real-Time PCR System. Standard thermal cycling conditions were applied, followed by a Melt curve analysis to confirm the specificity of the amplification. All reactions were run in triplicate, and gene expression was normalized to β-actin. The 2^−ΔΔCt^ method was used to calculate relative gene expression. The primer sequences for this study can be found in Table S3.

### Western blotting and immunoprecipitation

To extract total protein, BMSCs were harvested post-treatment and rinsed with cold PBS. Lysis was performed on ice using RIPA buffer (Beyotime, ST506) supplemented with 1% phenylmethylsulfonyl fluoride. The resulting lysates were collected, mixed with loading buffer, and thermally denatured at 100 °C for 15 min. Protein samples were then resolved by SDS-PAGE (10% or 12.5% gels) and electro-transferred onto PVDF membranes (Bio-Rad). To minimize background signal, membranes were blocked with a protein-free fast blocking buffer (Epizyme, Shanghai, China) at ambient temperature for 30 min. Subsequently, the membranes were probed with primary antibodies at 4 °C overnight.

For detection, a biotin-streptavidin amplification system was employed. After washing with Tris-buffered saline-Tween 20, membranes were incubated with a biotinylated secondary antibody for 30 min, followed by a 30 min incubation with horseradish peroxidase-conjugated streptavidin. Immunoreactive bands were visualized using a chemiluminescent substrate (160072, Saimike) and captured on a ChemiScope 6200 system. Band intensity was quantified via ImageJ software.

For co-immunoprecipitation assays, cell lysates were prepared using RIPA buffer containing a cocktail of protease and phosphatase inhibitors (Bimake). Protein G magnetic beads (NEB, S1430) were pre-equilibrated with lysis buffer. The lysates were then incubated overnight at 4 °C with specific primary antibodies (targeting HNRNPA1 or CNOT1), with IgG used as a negative control. The antibody–antigen complexes were captured by incubating with the pre-washed magnetic beads at 4 °C for 1 h. Finally, the bead-bound complexes were washed, eluted in lysis buffer, and subjected to immunoblotting analysis as described above.

### RNA immunoprecipitation

RNA immunoprecipitation was conducted using the BeyoRIP™ RIP Assay Kit (Protein A/G agarose). Briefly, cells were lysed in RNA immunoprecipitation lysis buffer and cleared by centrifugation at 14,000 *g* at 4 °C for 15 min. Anti-HNRNPA1 antibody (11176-1-AP, ProteinTech, Wuhan, China) or control rat IgG was incubated with Protein A/G magnetic beads at room temperature for 30 min with gentle rotation. The antibody–bead complexes were then incubated with pre-cleared cell lysates at 4 °C overnight under continuous rotation. After washing, RNA was extracted from the immunoprecipitates using TRIzol reagent and subsequently analyzed by reverse transcription followed by PCR.

### Detection of intracellular iron ions, reactive oxygen species (ROS), and malondialdehyde

Intracellular Fe^2+^ levels were measured using a ferrous ion fluorescence detection kit (S1070S, Beyotime, Shanghai, China). Malondialdehyde content, indicative of lipid peroxidation, was assessed with a commercial lipid peroxidation detection kit (S0043S, Beyotime, Shanghai, China). Both Fe^2+^ and malondialdehyde signals were visualized by confocal laser microscopy. Cellular ROS levels were determined using a superoxide anion-sensitive fluorescent probe kit (S0064S, Beyotime, Shanghai, China), with fluorescence intensity quantified by a microplate reader.

### Transmission electron microscopy

To investigate mitochondrial morphological changes, BMSCs were collected and pre-treated with 3% glutaraldehyde. The specimens were subsequently fixed in 1% osmium tetroxide, dehydrated in a series of graded acetone solutions, infiltrated with Epon 812 for extended periods, and then embedded. Semi-transparent sections were stained with methylene blue, and ultra-thin sections were cut using a diamond knife and further stained with uranyl acetate and lead citrate. The sections were examined using a JEM-1400-FLASH transmission electron microscope.

### Animal experiments

All animal procedures were approved by the Institutional Ethics Committee of Chongqing Medical University (IACUC-CQMU-2025-07043) and conducted in accordance with the National Institutes of Health Guidelines for the Care and Use of Laboratory Animals. Male Sprague–Dawley rats (8 weeks old, ∼200 g) were obtained from the Experimental Animal Center of Chongqing Medical University. The animals were housed under specific pathogen-free conditions at a temperature of 22 ± 2 °C, humidity of 50% ± 10%, and a 12 h/12 h light/dark cycle, with free access to standard chow and filtered water.

After a 1-week acclimatization period, rats were randomly allocated into five experimental groups (*n* = 5 per group): Control (normal diet), CKD model (0.75% adenine + 1.03% high-phosphorus diet), CKD + Betaine (2% w/w in diet), CKD + AAV-ABCG1, and CKD + Betaine + AAV-shABCG1. CKD was induced by daily oral gavage of 0.75% adenine suspension (250 mg/kg) for 3 weeks, followed by a high-phosphorus diet (1.03% phosphorus) from weeks 4–8. Control rats received a standard diet throughout the experimental period. For gene delivery interventions, rats in the AAV-ABCG1 and AAV-shABCG1 groups received a single tail-vein injection of AAV vectors (2 × 10^12^ viral genomes in 200 μL saline). Betaine treatment was administered via a diet supplemented with 2% (w/w) betaine for 4 weeks.

At the end of the experimental period, rats were anesthetized with intraperitoneal pentobarbital sodium (50 mg/kg). Blood and tissue samples (kidney, femur, and tibia) were collected for downstream analyses. Subsequently, euthanasia was performed in a CO_2_ chamber at a displacement rate of 60% of the chamber volume per minute, followed by continued exposure for at least 2 min after the cessation of breathing to ensure death.

### Histological evaluation and staining

Collected rat femurs were fixed in 4% paraformaldehyde for 3 days, followed by decalcification in EDTA solution (pH 7.2) for 21 days. Tissues were then dehydrated through a graded ethanol series, cleared in xylene, and embedded in paraffin. Sections of 5 μm thickness were cut, deparaffinized, rehydrated, and stained with hematoxylin and eosin or Masson's trichrome according to standard protocols. Stained sections were imaged under a light microscope (Nikon Ci-L, Tokyo, Japan), with at least three representative fields captured per section for analysis.

### Micro-computed tomography

To assess bone microarchitecture, excised rat femurs were subjected to high-resolution imaging using a SkyScan 1276 micro-CT scanner (Bruker, Germany). The resulting raw data were reconstructed into 3D images via NRecon software (version 1.7.4.2). Subsequently, quantitative morphometric analysis was carried out using the manufacturer's standard analysis suite to determine key trabecular indices, including bone mineral density, bone volume fraction, as well as trabecular thickness, trabecular separation, and trabecular number.

### Isolation and culture of primary rat BMSCs

Primary BMSCs were harvested from the long bones (femur and tibia) of 6-week-old male Sprague–Dawley rats. Under aseptic conditions, the epiphyses were excised, and the marrow cavities were flushed with complete DMEM using a 21 gauge needle attached to a 10 mL syringe. To ensure a single-cell suspension, the marrow eluate was filtered through a 70 μm mesh to eliminate bone debris, followed by centrifugation at 1000 rpm for 5 min. The resulting cell pellet was resuspended and plated into T25 flasks, then incubated in a humidified atmosphere at 37 °C with 5% CO_2_. Non-adherent populations were removed by refreshing the medium every three days. Subculturing was performed once the adherent monolayer reached approximately 80% confluence. Cell identity was subsequently verified via flow cytometry (Fig. S1).

### Statistical analysis

Data were presented as mean ± standard error of the mean. All experiments were independently repeated at least three times. Image quantification for Western blotting, ALP staining, Alizarin red S staining, and fluorescence imaging was performed using ImageJ software. Statistical analyses were conducted with GraphPad Prism 9.5.0. Before parametric analyses, data distribution was assessed for normality using the Shapiro–Wilk test. For comparisons between two groups, the two-tailed Student's *t*-test was applied; for comparisons among three or more groups, one-way ANOVA with appropriate post-hoc tests was used. If data did not conform to a normal distribution, nonparametric tests were applied accordingly. A *p*-value < 0.05 was considered statistically significant. This study was conducted and reported in accordance with the ARRIVE guidelines 2.0.

## Results

### CKD-related bone disease is associated with intrinsic BMSC osteogenic dysfunction

We first established a CKD rat model using a 0.75% adenine plus 1% phosphate diet to model the pathological milieu of CKD-MBD. Kidney histology verified prominent renal injury (Fig. 1A). Femoral analysis revealed clear skeletal abnormalities, with hematoxylin–eosin and Masson staining indicating disorganized and poorly mineralized bone matrix in CKD rats compared with controls (Fig. 1B). To directly assess osteoprogenitor competence in this pathological setting, we isolated primary BMSCs from control and CKD rats and induced osteogenesis *ex vivo*. BMSCs from CKD animals exhibited reduced RUNX2 and OPN expression and showed markedly lower ALP activity and mineralization capacity than controls (Fig. 1C–E). These findings indicate that CKD is associated with an intrinsic defect in BMSC osteogenic differentiation.

**Figure 1:**
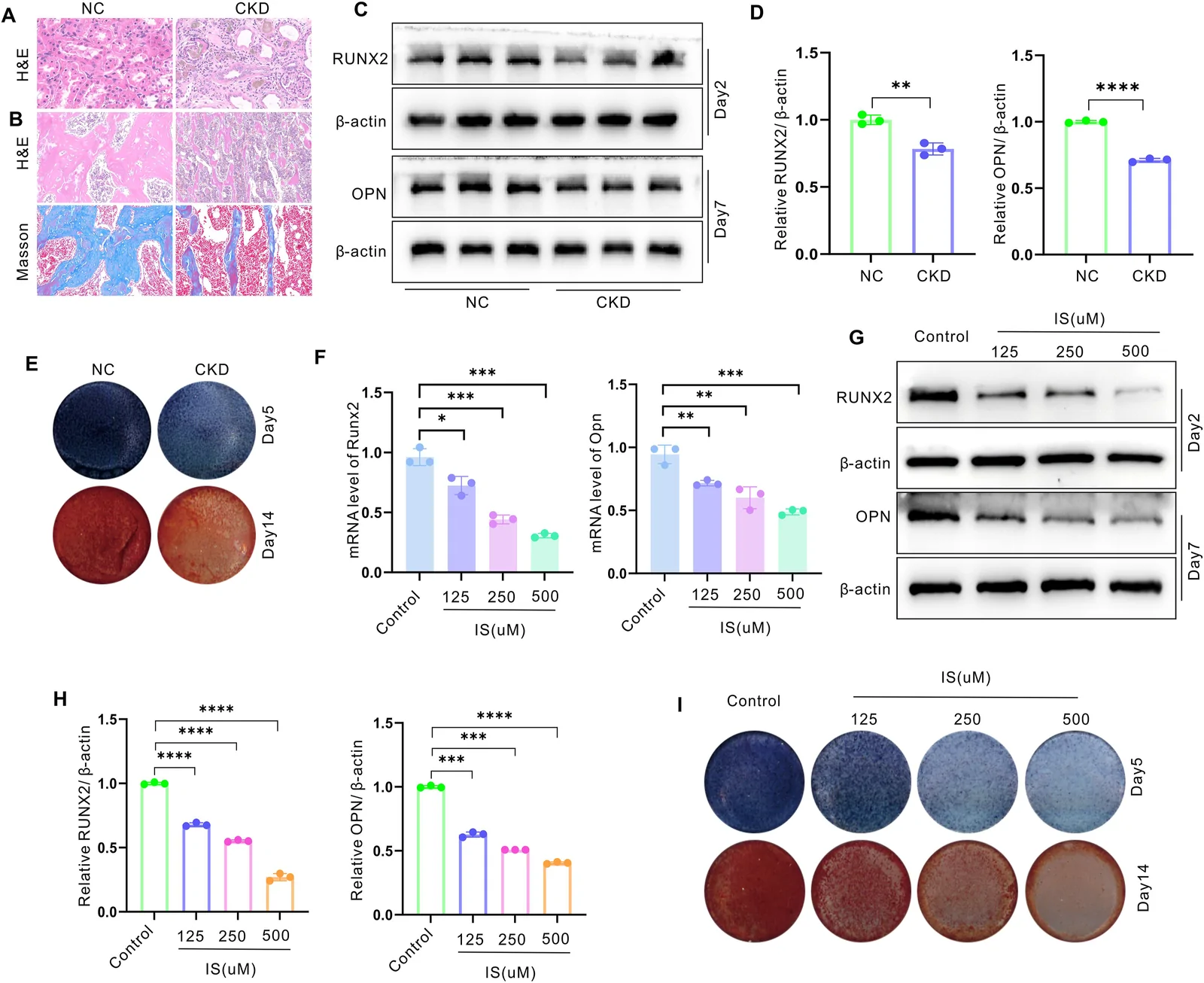
Indoxyl sulfate (IS) recapitulates the intrinsic osteogenic defects of bone marrow-derived mesenchymal stem cells (BMSCs) observed in chronic kidney disease (CKD) rats. **(A)** Representative hematoxylin–eosin-stained kidney sections from control and CKD rats (*n* = 5 per group). Scale bar, 50 μm. **(B)** Representative images of femoral bone tissue sections from Control and CKD rats stained with hematoxylin–eosin and Masson's trichrome (*n* = 5 per group). Scale bar, 50 μm. **(C, D)** Immunoblotting analysis (C) and quantification (D) of RUNX2 and OPN protein levels in primary BMSCs isolated from control and CKD rats after osteogenic induction for 2 or 7 days. **(E)** Representative images of alkaline phosphatase (ALP) staining (day 5) and Alizarin red S staining (day 14) of primary BMSCs from control and CKD rats. **(F)** mRNA expression levels of *Runx2* and *Opn* in BMSCs treated with increasing concentrations of IS (0, 125, 250, 500 μM) for 2 or 7 days, as determined by quantitative real-time PCR (*n* = 3). **(G, H)** Immunoblotting analysis (G) and quantification (H) of RUNX2 and OPN protein levels in BMSCs treated with increasing concentrations of IS (0, 125, 250, 500 μM) for 2 or 7 days (*n* = 3). **(I)** Representative images of ALP (day 5) and Alizarin red S (day 14) staining of BMSCs treated with the indicated concentrations of IS during osteogenic induction. Data were presented as mean ± standard deviation. ∗*p* < 0.05, ∗∗*p* < 0.01, ∗∗∗*p* < 0.001, and ∗∗∗∗*p* < 0.0001. NC, normal control group; Control, untreated BMSCs.

IS is a representative uremic toxin that accumulates in CKD and correlates with bone loss; we hypothesized that IS might be a primary driver of this BMSC dysfunction. To test this, we treated normal BMSCs with varying concentrations of IS. Consistent with our *in vivo* observations, IS treatment resulted in a concentration-dependent suppression of osteogenic differentiation, as evidenced by the down-regulated mRNA and protein expression of RUNX2 and OPN (Fig. 1F–H). Functionally, IS significantly inhibited ALP activity and attenuated mineralized nodule formation (Fig. 1I). Collectively, these data suggest that IS exposure mimics the intrinsic BMSC dysfunction observed in CKD animals, implicating IS as a critical contributor to ROD pathogenesis.

### Untargeted metabolomics identifies serum betaine depletion as a key metabolic signature of ROD

To systematically explore the metabolic perturbations underlying ROD, we performed untargeted metabolomic profiling on serum samples from ROD patients and healthy controls (Fig. 2A). Multivariate statistical analysis was employed to discriminate between the groups. Orthogonal partial least squares discriminant analysis (OPLS-DA) plots under both positive (Fig. 2B) and negative (Fig. 2C) ion modes revealed a clear separation between ROD patients and controls, indicating distinct metabolic phenotypes. To ensure the robustness of our model and rule out overfitting, we performed permutation tests (*n* = 200), which confirmed the validity of the OPLS-DA models in both ionization modes (Fig. 2D and E). To pinpoint the critical drivers of this distinction, we analyzed the differential metabolites. Notably, in the positive ion mode, betaine was identified as the most significantly down-regulated metabolite. Fold-change analysis (Fig. 2F) and volcano plots (Fig. 2G) confirmed that betaine was markedly down-regulated in ROD patients compared with controls (fold change = 0.19; Log_2_ fold change = −2.39; *p* < 0.001).

**Figure 2:**
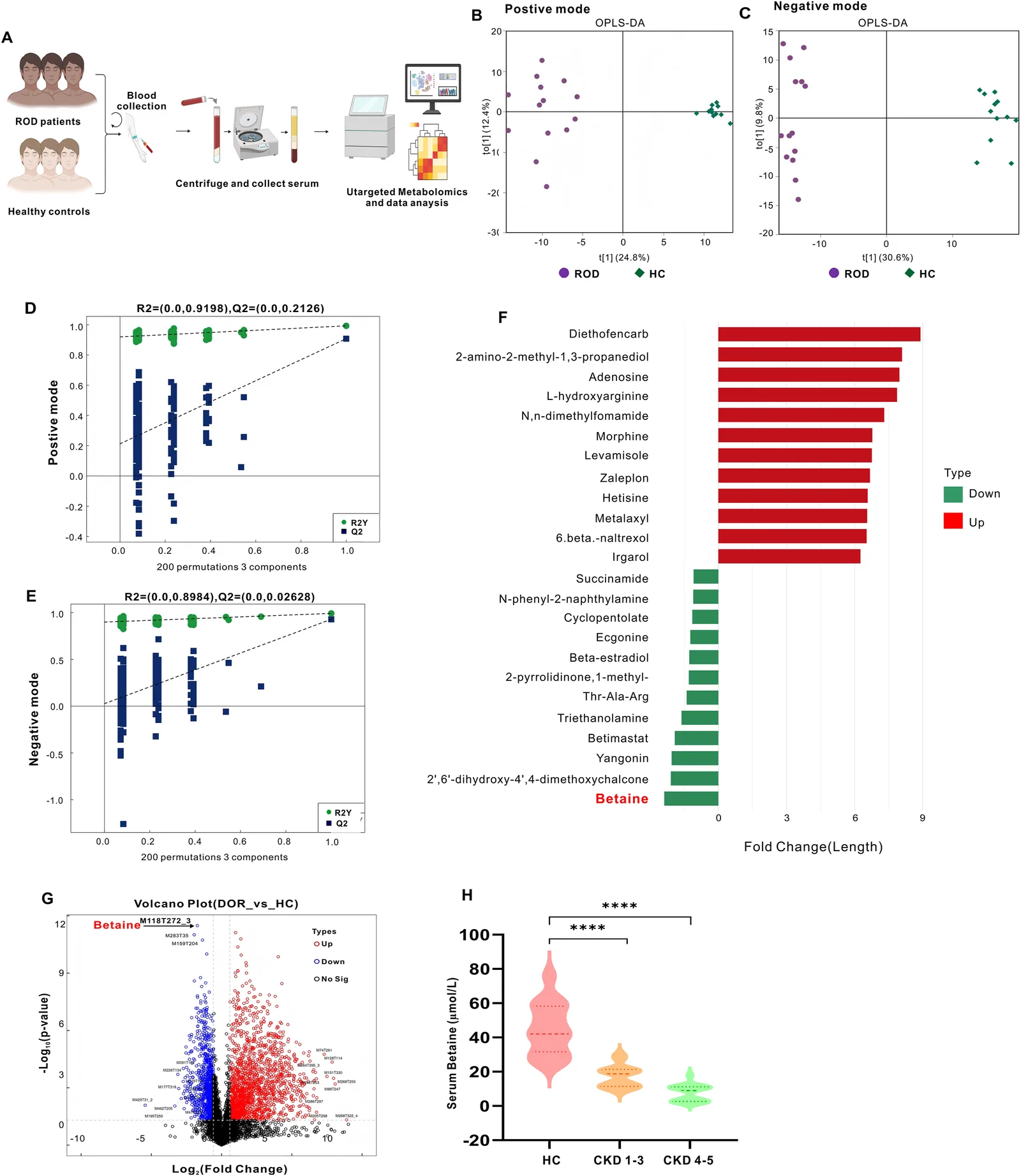
Metabolomics profiling identifies betaine as a key metabolite associated with renal osteodystrophy (ROD). **(A)** Schematic workflow of the metabolomic analysis performed on serum samples derived from ROD patients and healthy controls (HC) (ROD: *n* = 14; HC: *n* = 12). **(B, C)** Orthogonal partial least squares discriminant analysis (OPLS-DA) score plots showing distinct separation between ROD patients and controls in positive (B) and negative (C) ion modes. **(D, E)** Permutation tests (*n* = 200) validating the OPLS-DA models in positive (D) and negative (E) ion modes to rule out overfitting. **(F)** Bar chart showing the fold changes of differentially abundant metabolites between the control and ROD groups. **(G)** Volcano plot visualizing differentially expressed metabolites in ROD patients compared with controls. **(H)** Serum betaine levels across different chronic kidney disease (CKD) stages (stages 1–5) and healthy controls (HC) (CKD 1–3: *n* = 35; CKD 4–5: *n* = 35; HC: *n* = 30). Data were presented as mean ± standard deviation. ∗∗∗∗*p* < 0.0001.

We further measured serum betaine levels in healthy controls and patients with CKD at stages 1–5. Consistent with the omics data, we observed a progressive decline in serum betaine levels that paralleled the deterioration of renal function (Fig. 2H). Collectively, these data identify betaine depletion as a hallmark metabolic alteration in ROD, which is closely associated with disease progression.

### Betaine supplementation ameliorates ROD by restoring BMSC osteogenic function

To assess the therapeutic potential of betaine, we administered it to adenine-induced CKD rats following the establishment of renal and skeletal pathologies (Fig. 3A). Elevated serum betaine concentrations confirmed effective supplementation, which resulted in significant body weight recovery, reduced serum IS levels, and regulated calcium/phosphorus homeostasis (Table S4). Furthermore, betaine administration significantly improved renal function, as evidenced by reduced serum levels of blood urea nitrogen and creatinine compared with the untreated CKD group (Fig. 3B). Consistently, renal histopathological examination demonstrated that betaine treatment markedly attenuated kidney structural damage (Fig. 3C). We then assessed whether these renal benefits translated into skeletal protection. Micro-computed tomography (micro-CT) imaging revealed that betaine supplementation substantially preserved bone microarchitecture in CKD rats (Fig. 3D). Quantitative analysis confirmed that betaine significantly increased bone mineral density, bone volume fraction, trabecular thickness, and trabecular number, while reducing trabecular separation (Fig. 3G). Histological examination further validated these osteoprotective effects, showing enhanced bone matrix organization and mineralization (Fig. 3E and F). Moreover, immunohistochemical analysis indicated that betaine significantly up-regulated RUNX2 and OPN expression in bone tissue (Fig. 3H), suggesting enhanced osteogenic activity *in vivo*.

**Figure 3:**
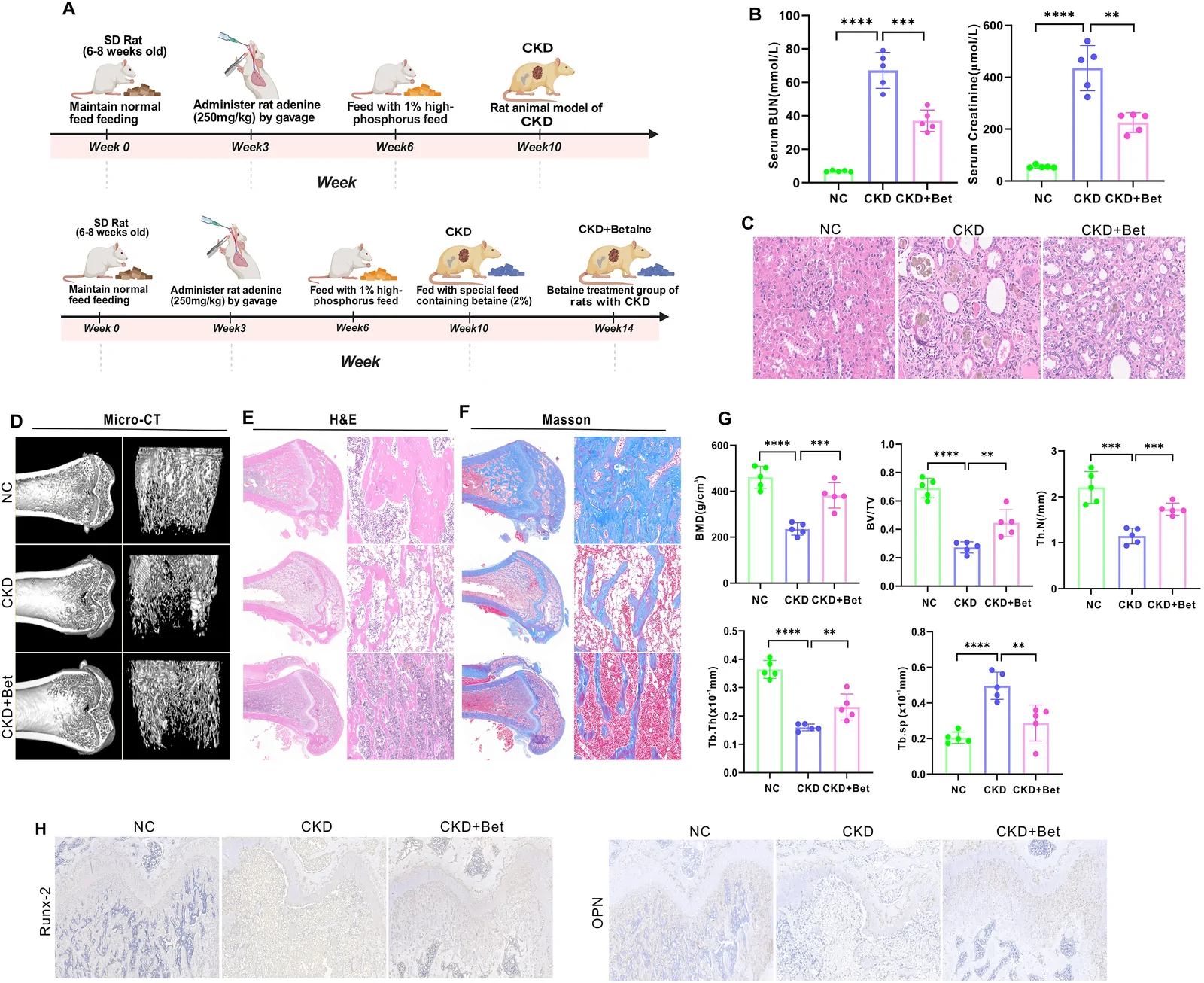
Betaine supplementation ameliorates renal injury and skeletal abnormalities in chronic kidney disease (CKD) rats. **(A)** Schematic of the *in vivo* experimental design. CKD was induced in rats via a 0.75% adenine +1% high-phosphate diet, followed by 8 weeks of betaine (1% w/v) or vehicle supplementation (*n* = 5). **(B)** Serum levels of blood urea nitrogen (BUN) and creatinine (Scr). **(C)** Representative hematoxylin–eosin-stained kidney sections. Scale bar, 50 μm. **(D)** Representative three-dimensional micro-CT reconstructions of distal femoral trabecular bone. Scale bar, 2 mm. **(E, F)** Representative histological images of femoral sections stained with hematoxylin–eosin (E) and Masson's trichrome (F). Scale bar, 50 μm. **(G)** Quantitative micro-CT analysis of bone mineral density (BMD), bone volume fraction (BV/TV), trabecular thickness (Tb.Th), trabecular number (Tb.N), and trabecular separation (Tb.Sp). **(H)** Representative immunohistochemistry images showing RUNX2 and OPN protein expression in femoral bone sections from each experimental group. Scale bar, 200 μm. Data were presented as mean ± standard deviation. ∗∗*p* < 0.01, ∗∗∗*p* < 0.001, and ∗∗∗∗*p* < 0.0001. Bet, betaine.

To determine whether the improved bone phenotype was associated with restoration of osteoprogenitor function, we isolated primary BMSCs of the experimental rats. After *in vitro* osteogenic induction, BMSCs from betaine-treated CKD animals exhibited significantly higher expression of RUNX2 and OPN compared with those from untreated CKD rats (Fig. 4A–C). Functional assays confirmed that betaine administration *in vivo* effectively rescued the osteogenic capacity of BMSCs, as demonstrated by enhanced ALP activity and mineralized nodule formation (Fig. 4D). These data suggest that betaine mitigates CKD-induced ROD, at least in part through restoring the intrinsic osteogenic differentiation potential of BMSCs.

**Figure 4:**
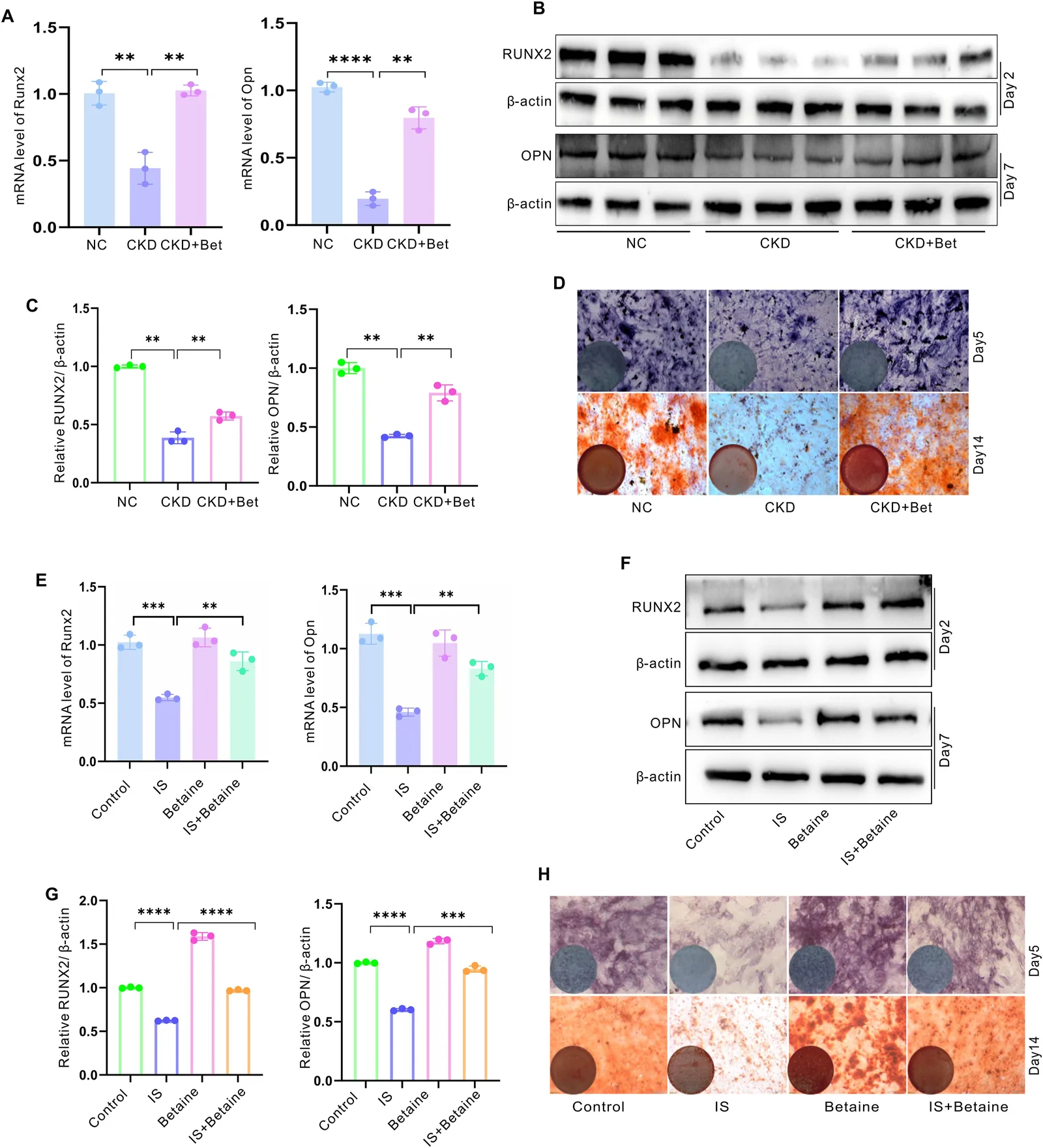
Betaine restores osteogenesis of bone marrow-derived mesenchymal stem cells (BMSCs) compromised by chronic kidney disease (CKD) or indoxyl sulfate (IS) exposure. **(A)** mRNA expression levels of *Runx2* and *Opn* in primary BMSCs isolated from rats in each group after 2 or 7 days of *in vitro* osteogenic induction, as determined by quantitative real-time PCR. **(B, C)** Immunoblotting analysis (B) and quantification (C) of RUNX2 and OPN protein levels in primary BMSCs isolated from rats in each group after 2 or 7 days of *in vitro* osteogenic induction. **(D)** Representative images of ALP (day 5) and Alizarin red S (day 14) staining of primary BMSCs from rats in each group. **(E)** mRNA expression levels of *Runx2* and *Opn* in BMSCs treated with IS (500 μM), betaine (5 mM), or both for 2 or 7 days, as determined by quantitative real-time PCR (*n* = 3). **(E, G)** Immunoblotting analysis (F) and quantification (G) of RUNX2 and OPN protein levels in BMSCs treated with IS (500 μM), betaine (5 mM), or both for 2 or 7 days. **(H)** Representative images of ALP and Alizarin red S staining in BMSCs treated with IS, betaine, or both during osteogenic induction. Scale bar, 100 μm. Data were presented as mean ± standard deviation. ∗∗*p* < 0.01, ∗∗∗*p* < 0.001, and ∗∗∗∗*p* < 0.0001. Bet, betaine; NC, normal control group; Control, untreated BMSCs.

We next examined whether betaine supplementation could directly counteract the detrimental effects of the uremic toxin IS on BMSCs. Co-treatment with betaine significantly alleviated IS-induced suppression of RUNX2 and OPN at both mRNA and protein levels (Fig. 4E–G). Functionally, betaine restored ALP activity and mineralized nodule formation in IS-treated BMSCs (Fig. 4H). Collectively, these results demonstrate that betaine acts directly on BMSCs to protect against IS-induced osteogenic impairment. To further define the specificity of this protection across the osteogenic lineage, we compared the effects of betaine on IS-treated C3H10T1/2 (multipotent mesenchymal progenitors), BMSCs, MC3T3-E1 (multipotent mesenchymal progenitors), and OCY454 (osteocytes). We found that while betaine exerted protective effects in all cell types, the rescue of osteogenic differentiation was most pronounced in BMSCs (Fig. S2). This stage-specific efficacy highlights BMSCs as the primary therapeutic target for betaine in ROD.

### ABCG1 is required for betaine-mediated rescue of BMSC osteogenesis and amelioration of ROD

To elucidate the molecular mechanisms underlying betaine-mediated protection, Kyoto Encyclopedia of Genes and Genomes (KEGG) analysis of metabolomic changes highlighted enrichment of the ABC transporter pathway (Fig. 5A). Corroborating this, transcriptomic profiling and Gene Set Enrichment Analysis (GSEA) revealed a marked up-regulation of ABC transporter-related genes in betaine-treated BMSCs compared with the IS-treated group (Fig. 5B). Among these candidates, ABCG1 emerged as a prominent differentially expressed target (Fig. 5C; Fig. S3A). Immunofluorescence staining of femoral sections further demonstrated that betaine treatment restored the co-expression of RUNX2 and ABCG1 *in vivo* (Fig. 5D). Consistently, IS treatment suppressed ABCG1 expression in BMSCs at both the mRNA and protein levels, whereas betaine supplementation effectively restored it (Fig. 5E and F; Fig. S3B).

**Figure 5:**
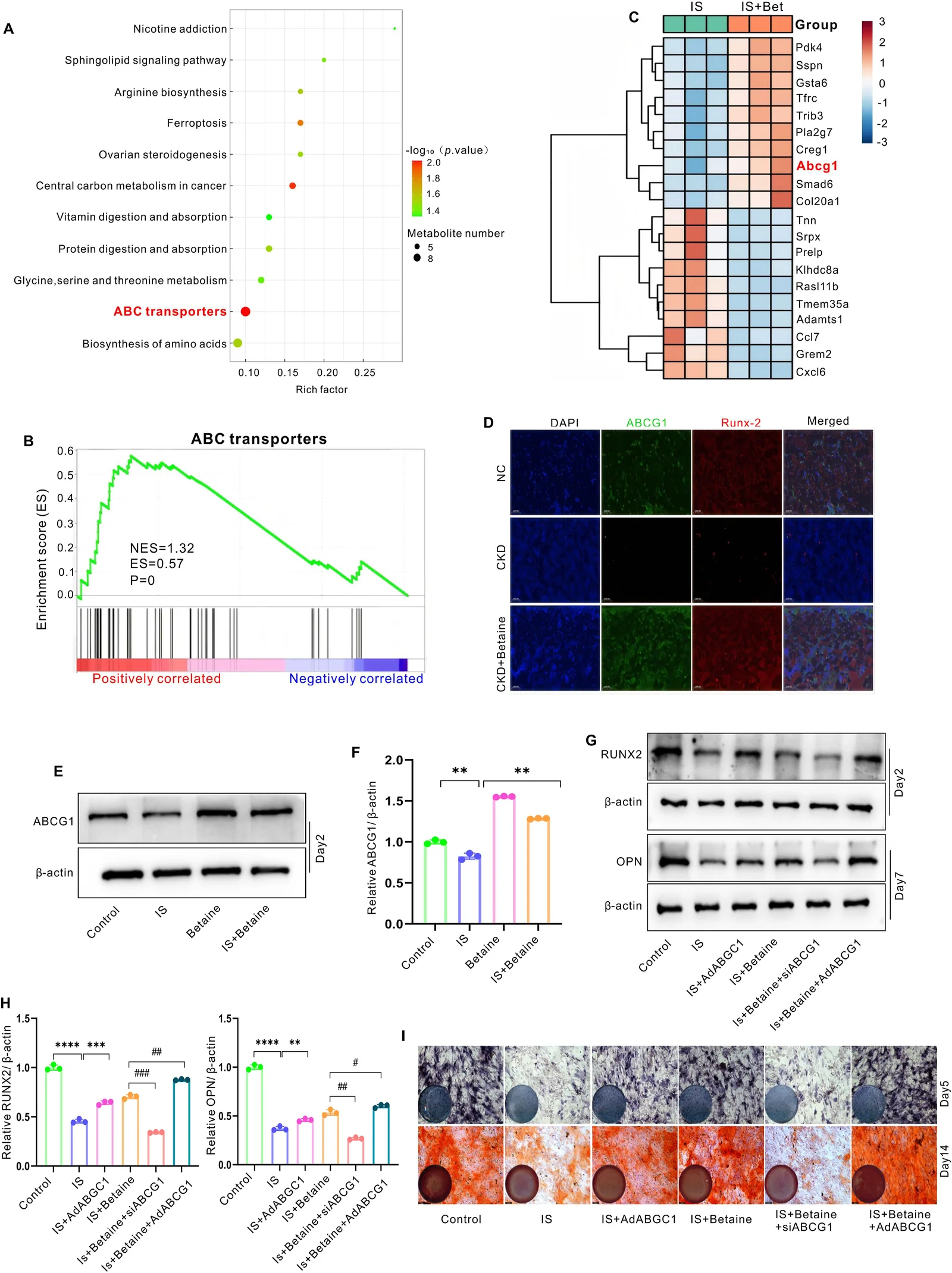
ABCG1 mediates the protective effects of betaine on bone marrow-derived mesenchymal stem cell (BMSC) osteogenesis. **(A)** Kyoto Encyclopedia of Genes and Genomes (KEGG) pathway enrichment analysis of serum metabolites from renal osteodystrophy patients. **(B)** Gene Set Enrichment Analysis (GSEA) plot of RNA-sequencing data from BMSCs treated with indoxyl sulfate (IS) or betaine, highlighting enrichment of ABC transporter pathways. **(C)** Heatmap of differentially expressed genes in BMSCs treated with betaine versus IS. **(D)** Immunofluorescence analysis of RUNX2 and ABCG1 expression in femoral bone sections from the betaine-treated group compared with the chronic kidney disease (CKD) group. Scale bar, 1 mm. **(E, F)** Immunoblotting analysis (E) and quantification (F) of ABCG1 in BMSCs treated with IS, betaine, or both (*n* = 3). **(G, H)** Immunoblotting analysis (G) and quantification (H) of RUNX2 and OPN protein levels in BMSCs treated with IS or betaine under conditions of ABCG1 overexpression or silencing. **(I)** Representative ALP (day 5) and Alizarin red S (day 14) staining of BMSCs with corresponding ABCG1 manipulations. Scale bar, 100 μm. Data were presented as mean ± standard deviation. ∗∗*p* < 0.01, ∗∗∗*p* < 0.001, ∗∗∗∗*p* < 0.0001, ^#^*p* < 0.05, ^##^*p* < 0.01, and ^###^*p* < 0.001. Bet, betaine; NC, normal control group; Control, untreated BMSCs.

Gain- and loss-of-function assays were employed to establish the functional requirement of ABCG1 in betaine-mediated protection. Overexpression of ABCG1 significantly alleviated the IS-induced suppression of RUNX2 and OPN, whereas ABCG1 knockdown abolished the protective effect of betaine (Fig. 5G and H; Fig. S3C). Functionally, ABCG1 overexpression restored ALP activity and mineralized nodule formation even under IS exposure; conversely, ABCG1 silencing negated the betaine-mediated recovery of osteogenic function (Fig. 5I).

We next investigated the physiological relevance of ABCG1 *in vivo* using AAV-mediated gene manipulation (Fig. 6A). Systemic analysis revealed that ABCG1 overexpression effectively rescued body weight and calcium/phosphorus homeostasis while reducing IS levels compared with the CKD group. Conversely, ABCG1 knockdown negated these systemic improvements in betaine-treated rats (Table S5). Remarkably, AAV-mediated overexpression of ABCG1 ameliorated renal dysfunction and pathological damage, whereas ABCG1 knockdown in betaine-treated rats attenuated the renoprotective effects of betaine (Fig. 6B and C). Skeletal assessment via micro-CT and histology revealed that ABCG1 overexpression significantly improved bone microarchitecture and matrix organization. In contrast, silencing ABCG1 blunted the skeletal benefits conferred by betaine (Fig. 6D–G). *Ex vivo* analysis further confirmed that BMSCs isolated from ABCG1-overexpressing rats exhibited restored RUNX2 and OPN expression and enhanced osteogenic capacity, while ABCG1 knockdown compromised the betaine-mediated rescue (Fig. 6H–J). These results identify ABCG1 as an essential effector of betaine, linking renal and skeletal improvement to the restoration of BMSC osteogenesis.

**Figure 6:**
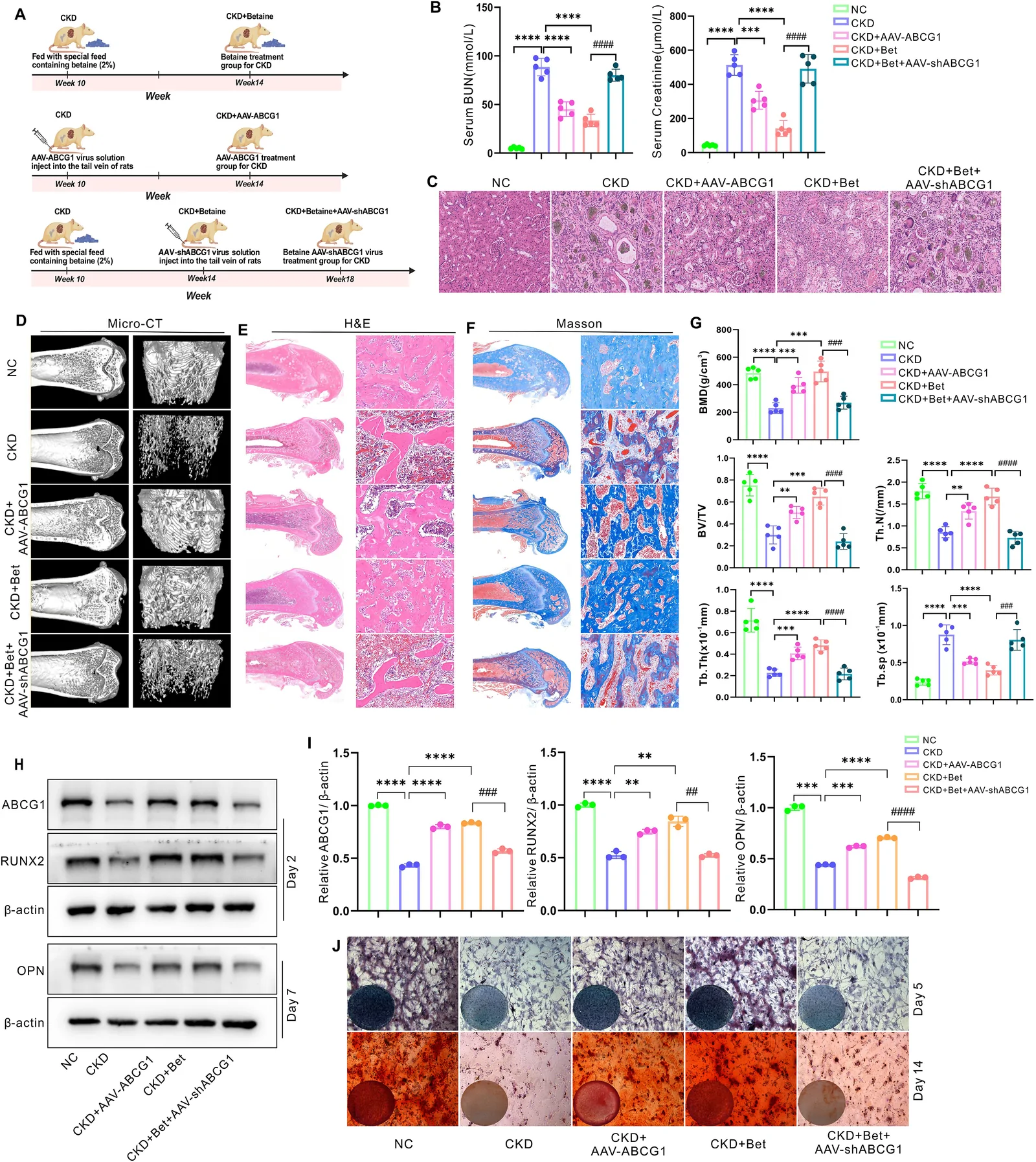
ABCG1 is required for the therapeutic efficacy of betaine against renal osteodystrophy *in vivo.***(A)** Schematic of the *in vivo* experimental design involving AAV-mediated ABCG1 manipulation (*n* = 5). **(B)** Serum blood urea nitrogen (BUN) and creatinine (Scr) levels. **(C)** Representative images of hematoxylin–eosin-stained kidney sections from the indicated experimental groups. Scale bar, 50 μm. **(D)** Representative three-dimensional micro-CT reconstructions of distal femoral trabecular bone. Scale bar, 2 mm. **(E, F)** Representative histological images of femoral sections stained with hematoxylin–eosin (E) and Masson's trichrome (F). Scale bar, 50 μm. **(G)** Quantitative micro-CT analysis of bone mineral density (BMD), bone volume fraction (BV/TV), trabecular thickness (Tb.Th), trabecular number (Tb.N), and trabecular separation (Tb.Sp). **(H, I)** Immunoblotting analysis (H) and quantification (I) of RUNX2 and OPN protein levels in primary bone marrow-derived mesenchymal stem cells (BMSCs) isolated from the *in vivo* experimental groups after *ex vivo* osteogenic induction. **(J)** Representative ALP (day 5) and Alizarin red S (day 14) staining of these primary BMSCs. Scale bar, 100 μm. Data were presented as mean ± standard deviation. ∗∗*p* < 0.01, ∗∗∗*p* < 0.001, ∗∗∗∗*p* < 0.0001, ^##^*p* < 0.01, ^###^*p* < 0.001, and ^####^*p* < 0.0001. Bet, betaine.

### ABCG1 promotes osteogenic differentiation by suppressing HMOX1-driven ferroptosis

To determine the molecular mechanism by which ABCG1 supports osteogenesis, we analyzed transcriptomic changes in betaine-treated BMSCs following ABCG1 knockdown. Differential expression analysis identified heme oxygenase-1 (HMOX1) as one of the significantly up-regulated genes (Fig. 7A and B). Validation experiments confirmed that ABCG1 overexpression suppressed IS-induced HMOX1 up-regulation, whereas ABCG1 knockdown attenuated the betaine-mediated inhibition of HMOX1 (Fig. 7C and D; Fig. S4A). To establish causality, we treated cells with the HMOX1 inhibitor zinc protoporphyrin (ZnPP). Pharmacological inhibition of HMOX1 significantly rescued the osteogenic defects induced by ABCG1 knockdown, restoring RUNX2 and OPN expression (Fig. 7E and F; Fig. S4B), as well as ALP activity and mineralized nodule formation (Fig. 7G).

**Figure 7:**
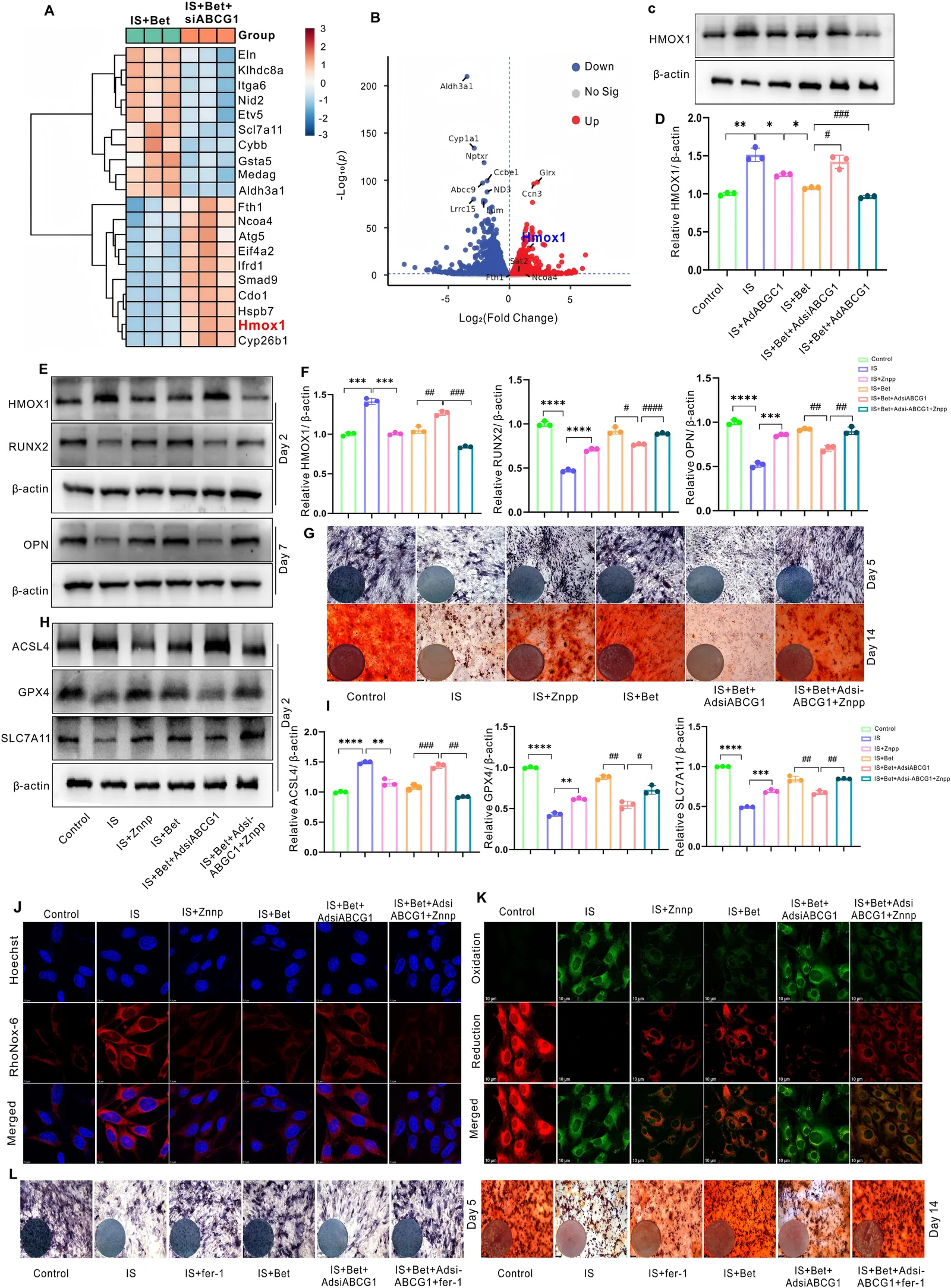
ABCG1 promotes osteogenic differentiation by suppressing HMOX1-driven ferroptosis. **(A, B)** Heatmap (A) and volcano plot (B) of differentially expressed genes (DEGs) in bone marrow-derived mesenchymal stem cells (BMSCs) upon ABCG1 knockdown under betaine treatment. **(C, D)** Immunoblotting analysis (C) and quantification (D) of HMOX1 protein levels in BMSCs under indicated treatments (*n* = 3). **(E, F)** Immunoblotting analysis (E) and quantification (F) of HMOX1, RUNX2, and OPN protein levels in BMSCs treated with indoxyl sulfate (IS) and betaine, with or without ABCG1 knockdown and/or the HMOX1 inhibitor ZnPP (*n* = 3). **(G)** Representative ALP and Alizarin red S staining under the indicated experimental conditions. **(H, I)** Immunoblotting analysis (H) and quantification (I) of the ferroptosis-related proteins ACSL4, GPX4, and SLC7A11 in BMSCs under the indicated treatment conditions (*n* = 3). **(J)** Representative fluorescence images showing intracellular ferrous iron (Fe^2+^) accumulation. Scale bar, 7.5 μm. **(K)** Analysis of lipid peroxidation levels via malondialdehyde (MDA) staining. Scale bar, 10 μm. **(L)** Representative images of ALP and Alizarin red S staining of BMSCs treated with the ferroptosis inhibitor Fer-1 to assess the rescue of osteogenic defects induced by ABCG1 silencing. Scale bar, 100 μm. Data were presented as mean ± standard deviation. ∗*p* < 0.05, ∗∗*p* < 0.01, ∗∗∗*p* < 0.001, ∗∗∗∗*p* < 0.0001, ^#^*p* < 0.05, ^##^*p* < 0.01, ^###^*p* < 0.001, and ^####^*p* < 0.0001. Bet, betaine; Fer-1, Ferrostatin-1; ZnPP, zinc protoporphyrin.

Given that HMOX1 is the rate-limiting enzyme in heme catabolism, its aberrant up-regulation leads to intracellular iron overload—a hallmark of ferroptosis. KEGG enrichment analysis of our transcriptomic data corroborated the involvement of the ferroptosis pathway (Fig. S4C). To provide definitive ultrastructural evidence for ferroptosis, we performed transmission electron microscopy. The analysis revealed that IS treatment induced characteristic ferroptotic changes in BMSCs, including shrunken mitochondria with increased membrane density. Notably, betaine treatment rescued this phenotype, whereas ABCG1 knockdown abolished the protective effect of betaine (Fig. S4D). Consistent with this, functional assays demonstrated that betaine treatment improved intracellular redox homeostasis, as evidenced by increased glutathione levels in IS-treated BMSCs; this protective effect was compromised by ABCG1 knockdown but restored by HMOX1 inhibition (Fig. S4E). Furthermore, betaine suppressed the up-regulation of the ferroptosis driver ACSL4 and restored the anti-ferroptotic markers GPX4 and SLC7A11 in an ABCG1-and HMOX1-dependent manner (Fig. 7H and I; Fig. S4F). Consistently, betaine significantly reduced intracellular ferrous iron (Fe^2+^) accumulation, ROS generation, and lipid peroxidation through the ABCG1–HMOX1 axis (Fig. 7J and K; Fig. S4G). Finally, the ferroptosis inhibitor Ferrostatin-1 alleviated IS-induced osteogenic impairment and rescued the loss of protection in ABCG1-silenced cells (Fig. 7L; Fig. S4H and S4I). Collectively, these data indicate that betaine preserves BMSC osteogenic differentiation by inducing ABCG1 expression, thereby suppressing HMOX1-mediated ferroptosis.

### Betaine up-regulates ABCG1 by inhibiting hnRNP A1 and disrupting hnRNP A1–CNOT1-mediated ABCG1 mRNA decay

Next, we performed proteomic profiling to identify the upstream regulators responsible for betaine-induced ABCG1 induction. This analysis revealed that hnRNP A1 was significantly down-regulated following betaine treatment (Fig. 8A–C). hnRNP A1 is a critical RNA-binding protein known to modulate mRNA stability by recruiting the CCR4–NOT deadenylase complex—scaffolded by CNOT1—to specific target transcripts for degradation. Based on this, we hypothesized that betaine enhances ABCG1 expression by interfering with hnRNP A1-mediated mRNA decay.

**Figure 8:**
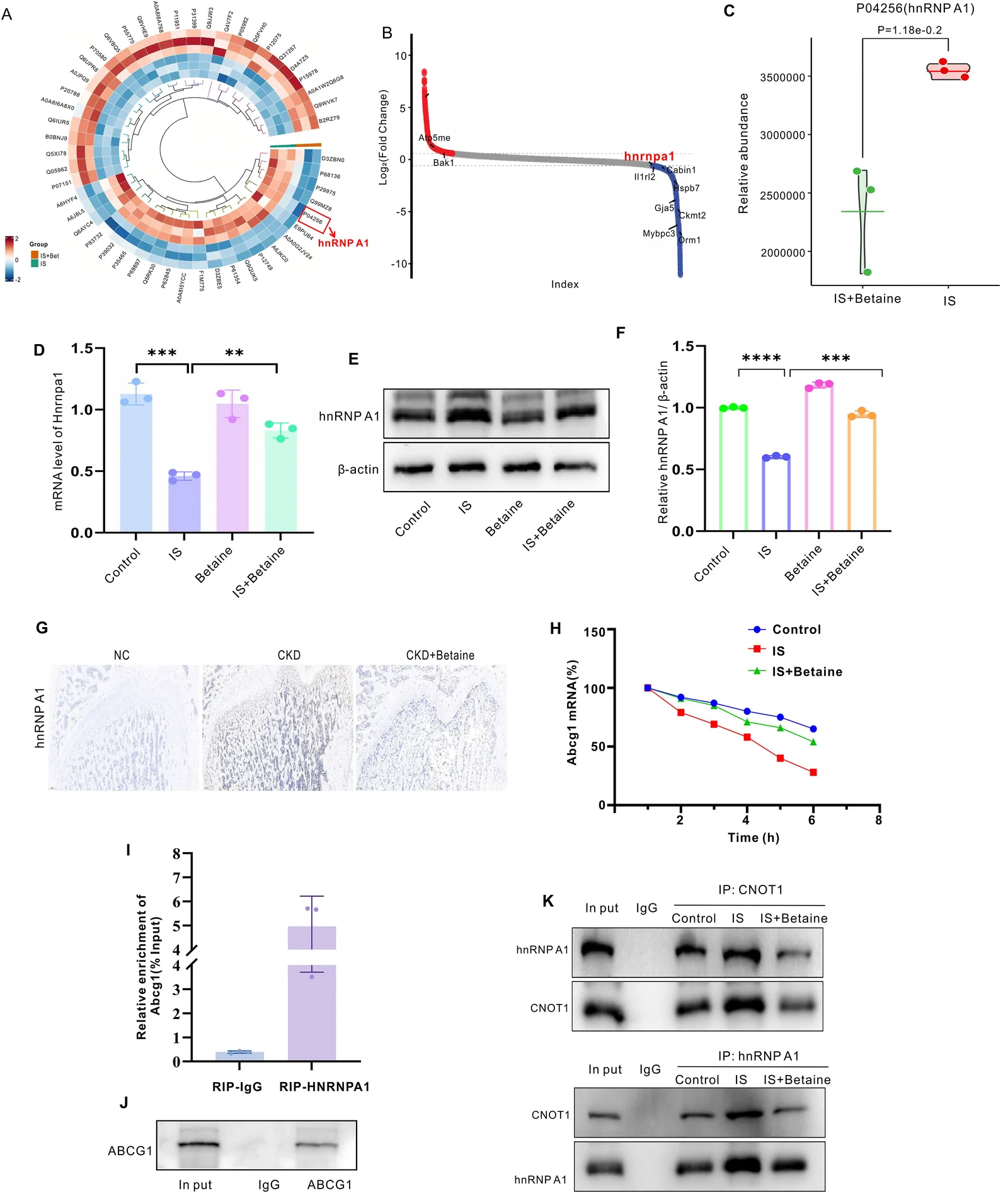
Betaine stabilizes *Abcg1* mRNA by inhibiting the hnRNP A1–CNOT1 axis. **(A**–**C)** Proteomic profiling of bone marrow-derived mesenchymal stem cells (BMSCs) treated with indoxyl sulfate (IS) and/or betaine. (A) Circular heatmap of differentially expressed proteins. (B) *P*-value–fold change scatter plot. (C) Scatter–violin plot showing the relative protein abundance of hnRNP A1 under different treatment conditions. **(D)** Quantitative real-time PCR analysis of *Hnrnpa1* mRNA expression levels in BMSCs treated with IS, betaine, or both (*n* = 3). **(E, F)** Immunoblotting analysis (E) and quantification (F) of hnRNP A1 protein levels in BMSCs treated with IS, betaine, or both (*n* = 3). **(G)** Representative immunohistochemistry images showing hnRNP A1 protein expression in femoral bone sections from the indicated experimental groups. Scale bar, 200 μm. **(H)** Analysis of *Abcg1* mRNA stability. BMSCs were pretreated with IS and/or betaine, followed by transcription inhibition with Actinomycin D (ActD, 5 μg/mL), and mRNA levels were quantified at the indicated time points (*n* = 3). **(I, J)** RNA immunoprecipitation assay. (I) Interaction between hnRNP A1 protein and *Abcg1* mRNA assessed by quantitative PCR. (J) Western blotting analysis of hnRNP A1 in input and immunoprecipitation (IP) samples. **(K)** Immunoprecipitation analysis of the protein–protein interaction between hnRNP A1 and CNOT1 under the indicated treatments. Data were presented as mean ± standard deviation. ∗∗*p* < 0.01, ∗∗∗*p* < 0.001, and ∗∗∗∗*p* < 0.0001. Bet, betaine.

Validation experiments confirmed that IS induced, whereas betaine suppressed, hnRNP A1 up-regulation at both the mRNA and protein levels (Fig. 8D–F). Consistently, immunohistochemical analysis showed reduced hnRNP A1 expression in the femoral bone tissue of betaine-treated CKD rats (Fig. 8G). To verify the post-transcriptional mechanism, we assessed mRNA turnover rates and found that betaine treatment significantly increased the half-life of *Abcg1* mRNA (Fig. 8H). Mechanistically, RNA immunoprecipitation assays demonstrated direct binding of hnRNP A1 to *Abcg1* mRNA (Fig. 8I and J). Furthermore, immunoprecipitation analysis revealed that IS promoted the recruitment of CNOT1 by hnRNP A1, whereas betaine treatment effectively disrupted this hnRNP A1–CNOT1 interaction (Fig. 8K). These findings support a model in which betaine restores ABCG1 levels via a dual post-transcriptional mechanism: by reducing the abundance of hnRNP A1 and by dismantling the hnRNP A1–CNOT1 complex, thereby protecting Abcg1 mRNA from targeted deadenylation and decay.

## Discussion

ROD represents a formidable clinical challenge in the management of CKD, with limited therapeutic options that can actively promote bone formation. The present study addresses this unmet clinical need by identifying betaine as a key metabolic mediator linking uremic toxin accumulation to BMSCss dysfunction, and by elucidating a novel post-transcriptional regulatory mechanism through which betaine exerts its osteoprotective effects. Our findings establish the hnRNP A1/ABCG1/ferroptosis axis as a central pathogenic pathway in ROD and highlight betaine supplementation as a promising active anabolic strategy.

Betaine, a critical quaternary amine alkaloid, functions as both an essential methyl donor and an osmolyte for maintaining cellular homeostasis. As the primary organ for betaine synthesis and reabsorption, the kidney is indispensable for maintaining systemic betaine homeostasis;^33^^,^^34^ consequently, renal impairment inevitably disrupts this equilibrium. In this study, our untargeted metabolomics analysis first identified a marked reduction in serum betaine levels in patients with ROD. Subsequent validation across different CKD stages confirmed that this depletion initiates in early CKD and exacerbates progressively with renal deterioration, a trend consistent with prior reports.^28^ Considering the established osteoprotective potential of betaine, we postulated that its systemic depletion contributes to the progression of bone pathology in CKD. Validating this hypothesis, our *in vivo* data demonstrated that betaine supplementation not only ameliorated renal function and bone anomalies in CKD rats but also revitalized the osteogenic differentiation capacity of BMSCs. Notably, because betaine also improved renal injury and reduced IS levels *in vivo*, its beneficial effect on bone may be attributable not only to a direct action on BMSCs but also, at least in part, to improvement of the overall uremic milieu. Mechanistically, our *in vitro* experiments further demonstrated that betaine rescued BMSCs from IS-induced osteogenic dysfunction, supporting a direct protective effect on BMSCs under controlled conditions independent of systemic factors. Collectively, these findings suggest that the bone-protective effect of betaine in CKD likely involves both direct cellular protection and indirect systemic improvement.

ABCG1, a prominent member of the ABC transporter superfamily, is fundamentally engaged in reverse cholesterol transport, mediating intracellular lipid efflux to maintain cholesterol homeostasis.^35^, ^36^, ^37^ Beyond its metabolic role, ABCG1 exhibits anti-inflammatory, antioxidant, anti-apoptotic, and immunomodulatory properties;^38^, ^39^, ^40^ however, its specific function within the osteoblast lineage remains elusive. In this study, our unbiased RNA-sequencing screen identified ABCG1 as a premier candidate significantly up-regulated by betaine. Validation assays demonstrated that the uremic toxin IS markedly suppressed ABCG1 expression in BMSCs, an effect effectively reversed by betaine intervention. Crucially, both *in vivo* and *in vitro* gain- and loss-of-function experiments confirmed that ABCG1 was the pivotal downstream effector mediating the osteoprotective actions of betaine. Although previous reports in C3H10T1/2 and C2C12 cells suggest that ABCG1 might negatively regulate osteogenesis via Wnt/β-catenin or AMPK pathways,^41^ our findings underscore a protective role. This discrepancy likely stems from differences in cell types and, more importantly, the specific pathological microenvironment. As a critical lipid transporter, ABCG1 orchestrates the distribution of phospholipids and polyunsaturated fatty acids within the plasma membrane,^42^ thereby preserving membrane fluidity and lipid raft integrity. Homeostasis of these membrane microdomains is not only essential for the activation of osteogenic signaling pathways (*e.g.*, BMP/Smad and Wnt/β-catenin),^43^^,^^44^ but its disruption may also lead to altered membrane lipid composition, thereby increasing cellular susceptibility to lipid peroxidation.

To further dissect the molecular machinery orchestrated by ABCG1, we performed transcriptomic profiling following ABCG1 silencing. This analysis highlighted HMOX1 as one of the most significantly differentially expressed genes regulated by ABCG1. As a key enzyme in the cellular stress response, HMOX1 catalyzes the degradation of heme into biliverdin, carbon monoxide (CO), and free iron (Fe^2+^). While physiologic HMOX1 activity is cytoprotective,^45^ accumulating evidence suggests that its sustained overactivation under pathological conditions accelerates heme degradation, leading to a drastic expansion of the intracellular labile iron pool.^46^, ^47^, ^48^ We postulated a mechanism where ABCG1 deficiency leads to the accumulation of peroxidation-susceptible lipid substrates, while the concurrent HMOX1 up-regulation fuels the process with catalytic iron (Fe^2+^). The convergence of these events ignites ferroptosis—an iron-dependent form of cell death characterized by lipid peroxidation and glutathione depletion. While ferroptosis has been implicated in various forms of osteoporosis,^49^, ^50^, ^51^ its specific occurrence and contribution to ROD—within the unique CKD milieu characterized by chronic inflammation and iron dysregulation—have not been previously reported in BMSCs. Our data robustly confirmed this hypothesis: IS treatment precipitated hallmark ferroptotic features in BMSCs, including lipid ROS accumulation, iron overload, glutathione depletion, and an ACSL4/GPX4 imbalance. Rescue experiments firmly established the causal chain: the protective effects of betaine were recapitulated by the HMOX1 inhibitor ZnPP or the ferroptosis inhibitor Ferrostatin-1. Conversely, ABCG1 silencing abolished betaine's protection and exacerbated HMOX1-mediated ferroptosis, a detrimental effect that was reversible by Fer-1 treatment. These findings establish the ABCG1–HMOX1–ferroptosis axis as a central driver of ROD.

Regarding the regulation of HMOX1 by ABCG1, several mechanisms are plausible. First, studies have indicated that ABC transporters can mediate heme export in certain cell types^52^; ABCG1 may facilitate the efflux of heme or its metabolites, thereby reducing substrate availability for HMOX1 and preventing excessive iron release. Second, similar to mechanisms observed in macrophages and other cells, ABCG1 could modulate HMOX1 expression via upstream signaling pathways such as NRF2,^53^^,^^54^ a similar regulatory network may exist in BMSCs. Collectively, our study demonstrates that betaine confers osteoprotection by restoring the ABCG1–HMOX1 axis to suppress ferroptosis. Although the precise molecular details of ABCG1-mediated HMOX1 regulation warrant further investigation, this study provides a novel perspective on the lipid–iron metabolic crosstalk driving uremic bone disease.

Finally, to elucidate the upstream molecular mechanisms underlying the up-regulation of ABCG1 by betaine, we performed a proteomic analysis and identified hnRNP A1 as a top candidate significantly modulated by betaine. Validation assays confirmed that IS markedly induced hnRNP A1 overexpression at both mRNA and protein levels in BMSCs, an effect effectively blunted by betaine intervention. Consistently, *in vivo* experiments demonstrated a significant reduction in hnRNP A1 abundance in the femoral tissues of betaine-treated CKD rats. hnRNP A1 is a versatile RNA-binding protein that plays a central role in mRNA metabolism, including splicing, transport, localization, and stability maintenance.^55^^,^^56^ While hnRNP A1 typically stabilizes mRNA under physiological conditions, it can functionally switch under specific stress stimuli to act as a molecular bridge that recruits degradation machinery, thereby accelerating transcript decay.^57^^,^^58^ Although the role of hnRNP A1 in neurodegenerative diseases and cancer has been extensively studied,^59^, ^60^, ^61^, ^62^ its function in skeletal biology and CKD-MBD remains unknown. In this study, we found that betaine treatment significantly extended the half-life of *Abcg1* mRNA, establishing that betaine enhances ABCG1 expression via a post-transcriptional mechanism.

Intracellular mRNA decay is primarily executed by the CCR4–NOT deadenylase complex.^63^^,^^64^ Our RNA immunoprecipitation assays revealed that IS promoted the physical binding of hnRNP A1 to *Abcg1* mRNA. Crucially, immunoprecipitation results demonstrated that IS significantly enhanced the interaction between hnRNP A1 and CNOT1, the core scaffold protein of the CCR4–NOT complex. This suggests that under IS stimulation, hnRNP A1 recruits the CNOT1 complex to *Abcg1* mRNA, thereby initiating its degradation. Notably, betaine intervention effectively disrupted this pathological hnRNP A1–CNOT1 interaction. It is well-established that IS exerts its osteotoxic effects primarily by activating the aryl hydrocarbon receptor (AhR). However, regarding the post-transcriptional regulatory network revealed in this study, the precise mechanism by which betaine down-regulates hnRNP A1 remains to be fully elucidated. Whether this effect is secondary to AhR pathway inhibition, stems from betaine's biochemical function as a major methyl donor, or involves other uncharacterized mechanisms warrants further investigation.

In conclusion, our study delineates a novel betaine–hnRNP A1–CNOT1–ABCG1 axis, providing a fresh post-transcriptional perspective on the molecular pathogenesis of ROD (Fig. 9). Nevertheless, IS was used in this study as a representative uremic toxin, whereas the uremic milieu in CKD is highly complex and contains multiple retained solutes, including protein-bound and small-molecule toxins. These toxins may exert additive or synergistic effects on BMSCs, and it remains unclear whether other major toxins, such as p-cresyl sulfate or ADMA, also converge on the hnRNP A1/ABCG1 pathway. Accordingly, whether betaine retains similar protective effects under multi-toxin conditions warrants further investigation.

**Figure 9:**
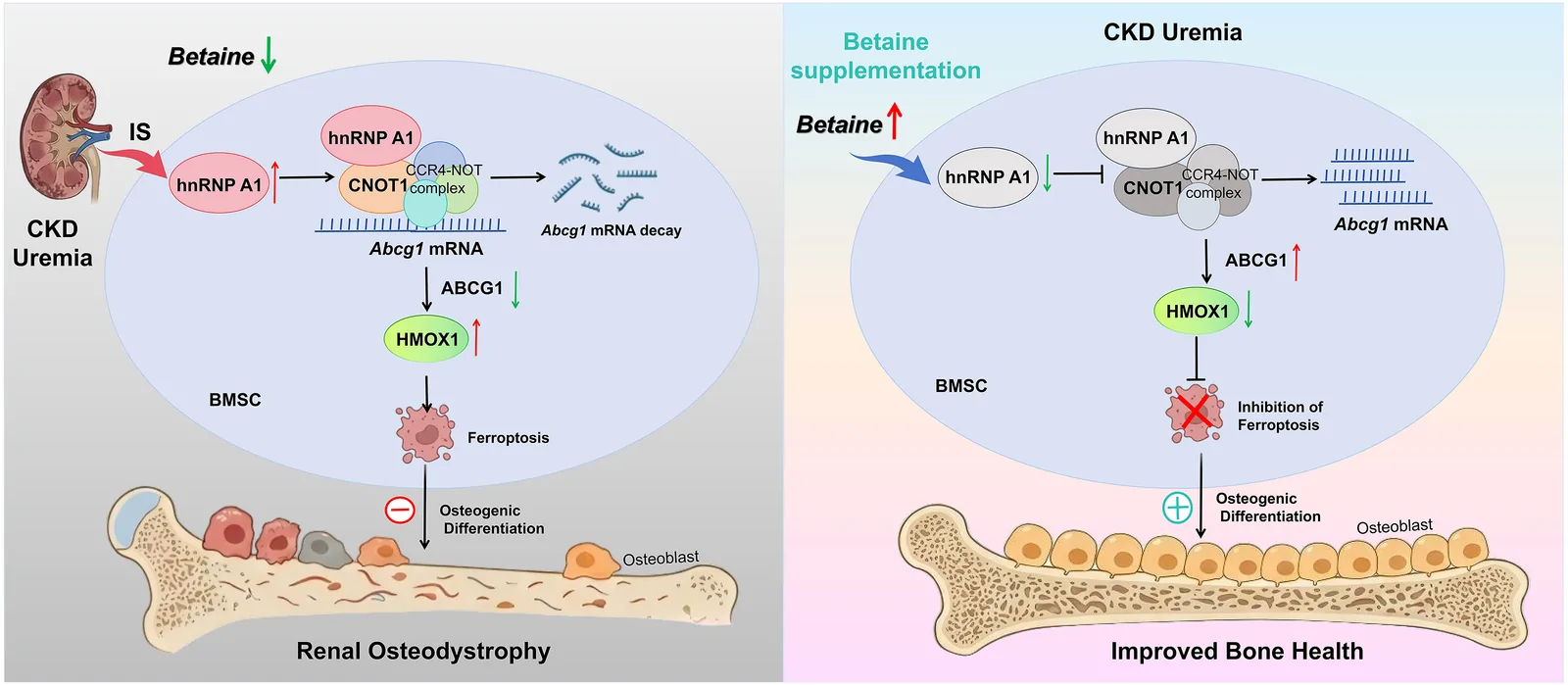
Schematic illustration of betaine-mediated protection against chronic kidney disease (CKD)-associated osteogenic impairment in bone marrow-derived mesenchymal stem cells (BMSCs). Under CKD uremic conditions, reduced betaine levels and indoxyl sulfate (IS) exposure promote hnRNP A1 up-regulation and CNOT1/CCR4-NOT-mediated *Abcg1* mRNA decay, leading to ABCG1 down-regulation and subsequent HMOX1-dependent ferroptosis in BMSCs. These changes ultimately impair osteogenic differentiation and contribute to renal osteodystrophy. Betaine supplementation suppresses this pathological process by restoring ABCG1 expression, attenuating HMOX1-dependent ferroptosis, and rescuing osteogenic differentiation, thereby improving bone health.

### Clinical translation and future perspectives

Our findings support a metabolic-molecular strategy for ROD and suggest that serum betaine may serve as a potential biomarker of disease progression in CKD-related bone disorder. As a clinically used nutrient supplement with a relatively favorable safety profile, betaine may have translational potential as an adjuvant therapy. Unlike conventional approaches focused mainly on passive mineral regulation, betaine may actively preserve BMSC osteogenic function by suppressing ferroptosis through the hnRNP A1/ABCG1 axis. In this study, betaine was administered at 2% (w/w) in the diet, corresponding to an estimated intake of approximately 1.5–2.0 g/kg/day in rats and a human equivalent dose of about 0.24–0.32 g/kg/day, or 14–19 g/day for a 60 kg adult. Although betaine is generally considered safe, this is a relatively high translated dose, and its long-term safety in CKD-MBD requires careful evaluation, particularly regarding gastrointestinal tolerance and possible effects on lipid metabolism. Future multicenter studies and randomized controlled trials are warranted to assess its efficacy, optimal dosing, and safety, as well as its potential relevance to the broader kidney–bone–vascular axis.

### Limitations

Despite the promising findings, several limitations warrant mention. First, while we utilized an adenine-induced rat model that closely mimics human CKD, species-specific differences in metabolism and bone turnover rates exist. Second, we focused primarily on IS as the representative uremic toxin; however, the uremic milieu is complex, and other toxins may also contribute to BMSCs ferroptosis through distinct mechanisms. Third, our study centered on the osteogenic differentiation of BMSCs (bone formation); further research is needed to determine whether this metabolic axis also modulates osteoclast function (bone resorption) or parathyroid hormone sensitivity in the context of CKD-MBD. Finally, while our data suggest betaine stabilizes Abcg1 mRNA via the hnRNP A1/CNOT1 axis, the lack of direct hnRNP A1 gain- and loss-of-function combined with mRNA half-life assays prevents definitive proof of a causal relationship, warranting future validation.

## Conclusion

In summary, our study establishes a critical pathogenic link in ROD extending from systemic metabolic deficiency to ferroptosis-driven stem cell failure. We demonstrate that progressive betaine depletion triggers the hnRNP A1/CNOT1-mediated decay of *Abcg1* mRNA. The consequent loss of ABCG1 triggers HMOX1-dependent ferroptosis, ultimately arresting the osteogenic differentiation of BMSCs. By bridging the gap between metabolic dysregulation and post-transcriptional control, this work not only elucidates the molecular etiology of uremic bone disease but also identifies the “betaine–ABCG1″ axis as a viable therapeutic target. These findings offer a promising anabolic strategy to address the unmet clinical need for restoring bone formation in ROD.

## CRediT authorship contribution statement

**Xinhang Li:** Writing – original draft, Visualization, Validation, Investigation. **Yi Xie:** Validation, Investigation, Formal analysis. **Wenyan Ren:** Visualization, Validation, Investigation. **Qing Liu:** Software, Methodology, Formal analysis. **Qiming Pang:** Software, Methodology, Formal analysis. **Wenting Liu:** Visualization, Methodology, Formal analysis. **Xin Li:** Visualization, Investigation. **Bin He:** Methodology, Formal analysis, Data curation. **Dongmei He:** Methodology, Investigation, Formal analysis. **Xinyue Wan:** Software, Methodology. **Jia Liu:** Visualization, Validation, Formal analysis. **Jie Cai:** Software, Methodology. **Baicheng He:** Resources, Data curation, Conceptualization. **Wei Huang:** Writing – review & editing, Supervision, Resources, Project administration, Conceptualization. **Fang He:** Writing – review & editing, Writing – original draft, Supervision, Project administration, Funding acquisition, Data curation, Conceptualization.

## Funding

This work was supported by the 10.13039/501100002858China Postdoctoral Science Foundation (No. 2024M763891), the 10.13039/501100001809National Natural Science Foundation of China (No. 82401850), the Young Excellence Scientific and Technological Talent Project of The First Affiliated Hospital of 10.13039/501100004374Chongqing Medical University (China) (No. ZYRC2022-02), the Qian De Young Seedling Talent Cultivation Project of the First Affiliated Hospital of Chongqing Medical University (China) (No. 2025QDPY-15), and the Bethune Public Welfare Foundation Project (China) (No. Z04J2025E170).

## Conflict of interests

The authors declared no competing interests.
