# Supplementary material for: Betaine alleviates renal osteodystrophy by stabilizing ABCG1 mRNA to inhibit HMOX1-mediated ferroptosis in BMSCs
Source: Genes Dis. 2026 Sep 4;14(2):102444. doi: 10.1016/j.gendis.2026.102444 (PMC13679308; doi:10.1016/j.gendis.2026.102444)
Supplement: Supplementary file 1 [file mmc1.docx]

**Table S1 Demographic and Clinical Characteristics of the Metabolomics Cohort**

| Characteristics | HC (n=12) | ROD(n=14) | *P* |
| --- | --- | --- | --- |
| Age, year  BMI, kg/m^2^  **SBP, mmHg**  **DBP, mmHg**  **ALB, g/L** | 40.3(9.4)  22.7(5.6)  114(8)  75(7)  45.2(3.5) | 51.7(16.1)  23.5(4.7)  141(13)  85(10)  38.7(4.5) | 0.07  0.84  ＜0.01  ＜0.01  ＜0.001 |
| **BUN, mmol/L** | 3.9(0.8) | 25.3(14.0) | ＜0.0001 |
| **Scr, umol/L** | 65.3(11.7) | 908.1(397.9) | ＜0.0001 |
| **UA, umol/L** | 320.3(66.6) | 444.8(141.3) | ＜0.01 |
| **eGFR, mL/min*1.73m^2^** | 123.1(16.0) | 6.8(4.5) | ＜0.0001 |
| **Ca, mmol/L** | 2.3(0.2) | 2.1(0.3) | 0.10 |
| **Pi, mmol/L** | 1.2(0.2) | 2.3(0.7) | ＜0.0001 |
| **PTH, pg/ml** | 51(12.4) | 854.6(710.7) | ＜0.0001 |
| **BMD( g/cm^2^ ) /T-score)** |  |  |  |
| **L1-4** | 1.1(0.1)/-0.14(0.4) | 0.8(0.1)/-2.2(1.5) | ＜0.0001 |
| **Femoral Neck** | 0.8(0.1)/-0.3(0.5) | 0.6(0.1)/-1.9(0.9) | ＜0.0001 |
| **Total Hip** | 0.9(0.1)/-0.2(0.6) | 0.6(0.1)/-2.8(0.7) | ＜0.0001 |

HC: Healthy control; ROD: Renal osteodystrophy, BMI: Body mass index, eGFR: estimated

glomerular filtration rate; SBP: Systolic blood pressure; DBP: Diastolic blood pressure.

ALB: albumin; BMD: Bone Mineral Density; L: Lumbar Spine.

**Table S2 Clinical Characteristics of the Validation Cohort**

| Characteristics | HC | CKD 1-3 stages | CKD4-5 stages |
| --- | --- | --- | --- |
| Age,year  BMI,kg/m^2^  **SBP,mmHg**  **DBP,mmHg** | 51.5(9.4)  22.8(3.9)  113(6)  65(8) | 53.6(16.1)^ns^  23.4(3.2)^ns^  136(15)^**^  82(10)^**^ | 63.2(13.8)^***^  22.9(4.6)^ns^  138(23)^**^  83(13)^**^ |
| **BUN,mmol/L** | 4.9(1.1) | 12.2(7.6)^****^ | 26.6(8.9)^****^ |
| **Scr,umol/L** | 69.1(15.8) | 193.6(138.3)^****^ | 807.7(205.7)^****^ |
| **UA,umol/L** | 310.8(59.5) | 423.3(134.3)^****^ | 434.9(130.7)^****^ |
| **eGFR,mL/min*1.73m^2^** | 113.7(11.5) | 59.5(29.3)^****^ | 9.2(6.0)^****^ |
| **Ca,mmol/L** | 2.4(0.2) | 2.1(0.23)^****^ | 1.5(0.4)^****^ |
| **Pi,mmol/L** | 1.2(0.1) | 1.4(0.5)^*^ | 2.7(0.4)^*^ |
| **PTH,pg/ml** | 42.1(12.3) | 112.2(80.2)^***^ | 341.4(201.9)^****^ |
| **IS, mg/L** | 0.55(0.1) | 8.4(1.8)^****^ | 30.7(9.8)^****^ |
| Betaine,**umol/L** | 44.4(15.0) | 18.14(7.9)^****^ | 3.8(4.5)^****^ |

HC: Healthy control; BMI: Body mass index, eGFR: estimated glomerular filtration rate; SBP: Systolic blood pressure;DBP: Diastolic blood pressure; IS: indoxyl sulfate. Ns: Not significant. *P < 0.05, **P < 0.01, ***P < 0.001, ****P < 0.0001 vs. The HC group.

**Table S3 primer sequences**

| Genes | Forward primer(5′→3′) | Reverse primer(5′→3′) |
| --- | --- | --- |
| β-actin | CCACCATGTACCCAGGCATT | CGGACTCATCGTACTCCTGC |
| Runx2 | GCCGGGAATGATGAGAACTA | GGACCGTCCACTGTCACTTT |
| Opn | TGCACCCAGATCCTATAGCC | CTCCATCGTCATCATCATCG |
| Abcg1 | TTCTCCATGCTGTTCCTCATGTT | GAAAACACTCATCTCCAGGGGAA |
| Hmox1 | AAGCCGAGAATGCTGAGTTCA | GCCGTGTAGATATGGTACAAGGA |
| Slc7a11 | GGAAGTCACGACCGAACAGT | ATTGCCCTGCAGGTAACCTC |
| Gpx4 | ATGAAAGTCCAGCCCAAGGG | GTGTAGGGGCACACACTTGT |
| Acsl4 | GGAAAGCAAACTGAAGGCGG | AATGGCCATGTCTGAAGGGG |
| Hnrnpa1 | ACTGTGTGGTAATGAGAGATCCA | TGGCAGCATCCACTTCTTCC |

**Table S4 **Effects of betaine supplementation in CKD rats****

| Variable | NC | CKD | CKD+betaine |
| --- | --- | --- | --- |
| Body weight, g | 482.80(32.43)^****^ | 203.81(22.42) | 385.75(20.79)^****^ |
| **BUN,mmol/L** | 6.94(0.43)^***^ | 67.18(10.71) | 37.65(6.39)^**^ |
| **Scr,umol/L** | 55.54(5.42)^****^ | 475.32(41.41) | 225.28(38.14)^****^ |
| **UA,umol/L** | 57.88(5.52)^**^ | 172.10(40.45) | 92.64(11.67)^**^ |
| **Ca,mmol/L** | 2.71(0.20)^****^ | 1.31(0.26) | 1.85(0.18)^**^ |
| **Pi,mmol/L** | 2.53(0.20)^****^ | 5.65(0.32) | 4.05(0.64)^***^ |
| **IS, mg/L** | 0.38(0.13)^***^ | 7.10(1.34) | 1.58(0.40)^***^ |
| Betaine,**umol/L** | 218.01(26.38)^****^ | 85.68(15.22) | 298.28(23.63)^****^ |

Data are presented as mean ± SD (n=5). **P < 0.01, ***P < 0.001, ****P < 0.0001 vs. the CKD group.

**Table S5 In vivo validation of ABCG1 requirement for Betaine efficacy**

| Variable | NC | CKD | CKD+AAV-ABCG1 | CKD+Betaine | CKD+Betaine+  AAV-shABCG1 |
| --- | --- | --- | --- | --- | --- |
| Body weight, g | 507.23(30.31)^****^ | 194.51(21.13) | 343.82(41.03)^***^ | 401.75(27.05)^****^ | 219.40(22.68)^####^ |
| **BUN,mmol/L** | 6.38(0.70)^****^ | 90.25(6.25) | 60.22(4.42)^****^ | 43.90(9.31)^****^ | 88.25(8.31)^####^ |
| **Scr,umol/L** | 74.42()6.25^***^ | 468.73(61.06) | 275.40(22.23)^**^ | 216.96(27.54)^***^ | 439.30(42.15)^####^ |
| **UA,umol/L** | 48.18(6.30)^***^ | 235.70(42.08) | 157.62(23.84)^*^ | 113.12(14.04)^***^ | 216.15(34.99)^###^ |
| **Ca,mmol/L** | 2.58(0.14)^****^ | 1.28(0.17) | 1.97(0.10)^***^ | 2.09(0.13)^****^ | 1.40(0.25)^###^ |
| **Pi,mmol/L** | 2.19(0.21)^***^ | 4.71(0.72) | 4.12(0.39)^ns^ | 3.22(0.60)^**^ | 4.55(0.10)^##^ |
| **IS, mg/L** | 0.32(0.06)^****^ | 7.8(0.72) | 4.41(0.42)^****^ | 2.31(0.70)^****^ | 5.60(0.53)^####^ |
| Betaine,**umol/L** | 189.33(24.74)^****^ | 59.73(10.9) | 54.64(27.78)^ns^ | 246.30(31.48^)****^ | 247.56(19.52)^ns^ |

Data are presented as mean ± SD (n=5). *P < 0.05, **P < 0.01, ***P < 0.001, ****P < 0.0001 vs. the CKD group. ^#^P < 0.05, ^##^P < 0.01, ^###^P < 0.001,^####^P < 0.0001 vs. the CKD+Betaine group. ns: no significant difference.

**Figure S1 Identification of primary BMSCs.** Flow cytometry analysis revealed a phenotype (CD105⁺/CD90⁺ ≥90%; CD45⁻/CD14⁻) consistent with the standard for primary BMSCs.

**Figure S2 Stage-specific protective effect of betaine on osteogenic differentiation under IS treatment. (A)** qRT-PCR analysis of Runx2 and Opn mRNA expression in C3H10T1/2, BMSCs, MC3T3-E1, and OCY454 cells. **(B)** Western blot analysis of Runx2 and OPN protein expression in the indicated cells. **(C)** Quantitative analysis of Western blot results. **(D**) Alizarin Red S staining and quantification of mineralized nodules in the indicated cells.Scale bar, 100 μm. Data are presented as mean ± SD. **P* < 0.05,***P* < 0.01, ****P* < 0.001.

**Figure S3** **ABCG1 is required for betaine-mediated rescue of BMSCs osteogenesis. (A)** Volcano plot visualizing differentially edifferentially expressed genes in BMSCs treated with Betaine versus IS. **(B)** qRT-PCR analysis of *Abcg1* mRNA expression levels in BMSCs treated with IS, Betaine, or both (n = 3). **(C)** qRT-PCR analysis of Runx2 and *Opn* mRNA expression levels in BMSCs treated with IS or Betaine under conditions of ABCG1 overexpression or silencing (n = 3). Data are presented as mean ± SD. ***p* < 0.01, ****p* < 0.001. ^#^*p* < 0.05, ^##^*p* < 0.01, ^###^*p* < 0.001.

**Figure S4. ABCG1 promotes osteogenic differentiation by suppressing HMOX1-driven ferroptosis. (A)** qRT-PCR analysis of *Hmox1* mRNA expression levels in BMSCs treated with IS or Betaine under conditions of ABCG1 overexpression or silencing(n = 3). **(B)** qRT-PCR analysis of *Hmox1*, *Runx2* and *Opn* mRNA expression levels in BMSCs treated with IS and Betaine, with or without ABCG1 knockdown and/or the HMOX1 inhibitor ZnPP (n = 3). **(C)** KEGG pathway enrichment analysis of differentially expressed genes in BMSCs across experimental groups. **(D)** Transmission Electron Microscopy (TEM) analysis of ultrastructural changes. **(E)** GSH concentration in culture supernatants of BMSCs sunder the indicated conditions (n = 3). **(F)** qRT-PCR analysis of Acsl4, Gpx4 and Scl7a11 mRNA expression levels in BMSCs treated with IS and Betaine, with or without ABCG1 knockdown and/or the HMOX1 inhibitor ZnPP (n = 3). **(G)** Representative fluorescence microscopy images of intracellular reactive oxygen species (ROS) levels detected by dihydroethidium (DHE) staining. Scale bar, 50 µm. **(H, I)** Immunoblotting analysis (H) and quantification (I) of RUNX2 and OPN protein levels in BMSCs treated with ferroptosis inhibitor Ferrostatin-1 (Fer-1) to assess the rescue of osteogenic defects induced by ABCG1 silencing. Data are presented as mean ± SD. **p* < 0.05, ***p* < 0.01, ****p* < 0.001, *****p* < 0.0001. ^##^*p* < 0.01, ^###^*p* < 0.001, ^####^*p* < 0.0001.

Bet, Betaine; Fer-1, Ferrostatin-1; ZnPP, zinc protoporphyrin.

**Figure S4. Effect of Fer-1 alone on basal osteogenic differentiation.** **(A)** qRT-PCR analysis of Runx2 and OPN mRNA levels in BMSCs treated with Control or Fer-1 alone.
**(B)** Western blot analysis of Runx2 and OPN protein levels in BMSCs treated with Control or Fer-1 alone at Day 2 and Day 7. **(C)** Quantification of RUNX2 and OPN protein levels from the Western blot in (B). **(D)** ALP staining (Day 5) and Alizarin Red S staining (Day 14) of BMSCs treated with Control or Fer-1 alone. Data are presented as mean ± SD. ns, no significance. Fer-1: Ferrostatin-1.
